# Cancer-associated histone mutation H2BG53D disrupts DNA–histone octamer interaction and promotes oncogenic phenotypes

**DOI:** 10.1038/s41392-020-0131-0

**Published:** 2020-03-06

**Authors:** Yi Ching Esther Wan, Tsz Chui Sophia Leung, Dongbo Ding, Xulun Sun, Jiaxian Liu, Lina Zhu, Tze Zhen Evangeline Kang, Du Yang, Yuchen Zhang, Jitian Zhang, Chengmin Qian, Michael Shing Yan Huen, Qing Li, Maggie Zi Ying Chow, Zongli Zheng, Junhong Han, Ajay Goel, Xin Wang, Toyotaka Ishibashi, Kui Ming Chan

**Affiliations:** 1 Department of Biomedical Sciences, City University of Hong Kong, Hong Kong, China; 2 Key Laboratory of Biochip Technology, Biotech and Health Centre, Shenzhen Research Institute of City University of Hong Kong, Shenzhen, China; 3 Division of Life Science, Hong Kong University of Science and Technology, Hong Kong, China; 4 State Key Laboratory of Protein and Plant Gene Research, School of Life Sciences and Peking-Tsinghua Center for Life Sciences, Peking University, Peking, China; 5 School of Biomedical Sciences, The University of Hong Kong, Hong Kong, China; 6 Ming Wai Lau Centre for Reparative Medicine, Karolinska Institutet, Hong Kong, China; 7 State Key Laboratory of Biotherapy and Cancer Center, West China Hospital, West China Medical School, Sichuan University, Sichuan, China; 8 Department of Molecular Diagnostics and Experimental Therapeutics, Beckman Research Institute at City of Hope Comprehensive Cancer Center, Duarte, CA USA

**Keywords:** Epigenetics, Cancer genetics

**Dear Editor**,

Recent studies from us and others have deciphered the clinical relevance of a variety of oncohistones^1^ in different diseases. The H3K27M and H3K36M mutant histones exert dominant-negative effects on methylation levels of histone H3K27 and H3K36 on wild-type histone protein in pediatric brain cancers^2–4^ and chondroblastoma,^5,6^ respectively. In contrast to H3K27M and H3K36M that act in *trans* to cause global reduction of methylation at the respective residues, the H3G34 mutations, including H3G34V/R and H3G34W/L found in pediatric high-grade glioma and giant cell tumor of the bone, affect the H3K36 methylation in *cis* and alter the H3K36 methylation on the nucleosome containing the H3G34 mutation(s). In addition to histone H3, two recent reports revealed another class of oncogenic mutation in histone H2B.^7,8^ The glutamic acid at position 76 of H2B was found to be replaced by lysine in multiple cancers, including breast and lung carcinomas. Cells expressing the H2BE76K mutation acquired oncogenic phenotypes as a result of aberrant gene expression associated with cancer pathways, an effect possibly due to the destabilization of the histone octamer by H2BE76K. Together, these studies indicated that missense mutations in histone genes impact gene expression and play a vital role in driving tumorigenesis by distinct mechanisms.

To explore whether there are additional mutations in histone genes that promote tumorigenesis, we analyzed The Cancer Genome Atlas database and identified a H2BG53-to-D missense mutation (glycine 53 to aspartic acid) in 10 out of 146 pancreatic ductal adenocarcinoma (PDAC) patients (Fig. 1a and Supplementary Fig. S1). Of note, the H2BG53D mutation was also found in glioblastoma multiforme and lung squamous cell carcinoma. To consolidate this finding, we performed targeted sequencing of the H2B genes in an independent cohort of 121 PDAC patient samples. Two out of 121 tumor samples from PDAC patients were found to harbor the H2BG53D mutation (Supplementary Fig. S2). Together, we identified 12 patients harboring the H2BG53D mutation in 267 PDAC cases (4.5%). Compared to *KRAS*^G12D/V^ mutations that are found in >90% of PDAC patients, the relatively low percentage of H2BG53D suggested that, instead of driving PDAC at early stage, the H2BG53D is an acquired subclonal mutation and could possibly enhance PDAC development in cooperation with other drivers.

**Fig. 1:**
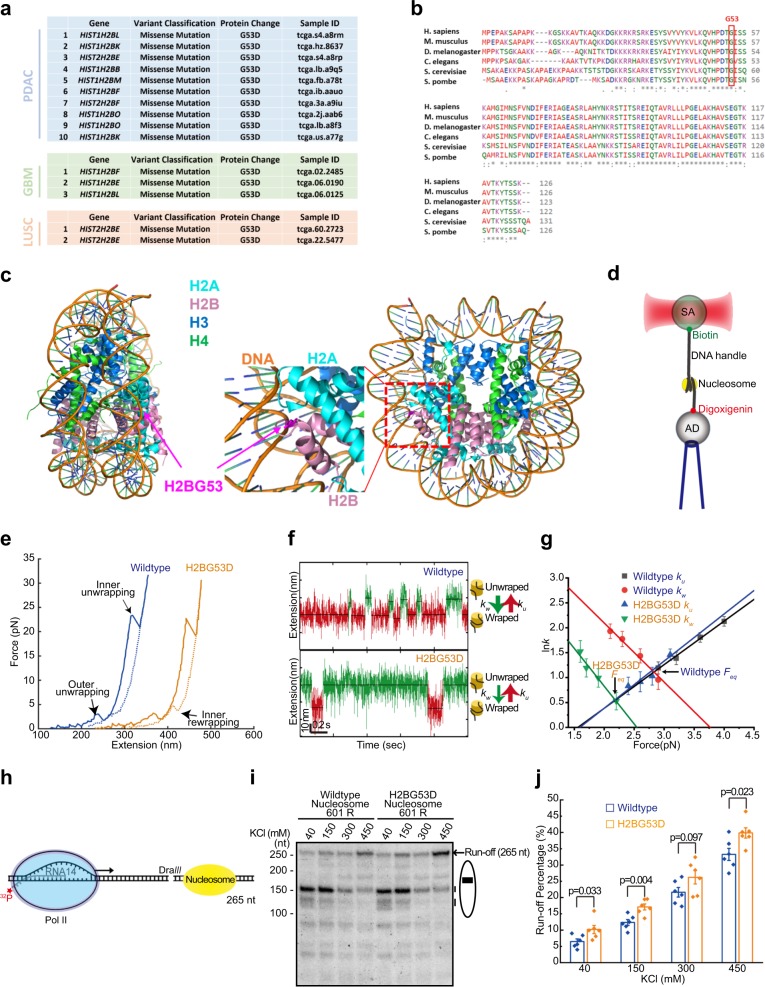
Cancer-associated H2BG53D mutation weakens the DNA–histone interaction and enhances transcription in vitro. **a** 10 out of 146 PDAC patients harbor the H2BG53D missense mutation. Glycine (G) at H2B position 53 is mutated to aspartic acid (D) in 1 of the 17 genes encoding histone H2B. The identities of the ten PDAC patients, three GBM patients, and two LUSC patients harboring the H2BG53D mutation are listed (Sample ID). All mutation data were downloaded from TCGA database. **b** Alignment of the amino acid sequences of H2B from different origins. Glycine at position 53 is highlighted in red rectangle. **c** Structural analysis of human nucleosome with the four core histone proteins labeled in different colors. G53 of H2B is highlighted in purple. **d** Schematic set-up of the optical tweezers used in the nucleosome studies. **e** Comparison of two typical force–extension curves of wild-type (blue) and H2BG53D (orange) nucleosome at 40 mM NaCl condition. The lines and the dots represent the unwrapping and the rewrapping events, respectively. **f** The representative nucleosome transition events between the wrapped state and the unwrapped state for the outer rip. **g** The relationship between the kinetic rate constant and external force. The equilibrium force *F*_eq_ was obtained from the point at which the folded rate (*k*_w_) equals the unfolded rate (*k*_u_). The error bars are the SEM of repeated measurements (*n* is shown in Supplementary Fig. 3d). **h** Experimental design of in vitro Pol II transcription elongation assay in the presence of a nucleosome. The arrow represents the direction of the transcription elongation. **i** In vitro transcription elongation assay gel in the presence of a wild-type or H2BG53D nucleosome. The ellipse represents the nucleosome and the black box is the nucleosome dyad region. **j** The relative run-off ratio of Pol II through the wild-type or the H2BG53D nucleosome (*n* = 6). The error bars represent SEM

Glycine at H2B position 53 is evolutionarily conserved (Fig. 1b), and structural analysis of the human nucleosome revealed that H2BG53 is located in close proximity to the double-stranded DNA near the DNA entry/exit site (Fig. 1c). The substitution of a neutrally charged glycine residue with a negatively charged aspartic acid at H2B-53 might affect the DNA–histone interaction. To dissect how H2BG53D affects DNA–histone interaction, single-molecule nucleosome pulling and holding assays were performed (Fig. 1d). As previously reported,^9^ two unwrap events were observed on the nucleosome pulling assay, the outer rip and the inner rip (Fig. 1e). At high-salt concentration (300 mM), the inner rips were mainly unwrapped around 15 pN in both wild-type and the H2BG53D nucleosomes, and both the rewrapping forces were close to 2 pN (Supplementary Fig. S3a, b). To quantify the effect of the mutation in the outer rips, we performed the nucleosome hopping experiment with the lower-salt concentration by holding a nucleosome tether (Fig. 1f and Fig. S3c, d). The unwrapping rate of the H2BG53D was similar to that in the wild-type nucleosome, but the G53D mutant decreased the wrapping rate by approximately four times (Fig. 1f). The equilibrium force (*F*_eq_), which is the wrapping and the unwrapping rates, was 2.83 pN in the wild-type nucleosome and was reduced to 2.17 pN in the H2BG53D nucleosome (Fig. 1g). To quantify the nucleosomal barrier in the outer rips, we extracted the free energy cost (Δ*G*). The Δ*G* in the wild-type nucleosome was 17.81 kJ/mol, and the Δ*G* in the H2BG53D was significantly reduced to 9.66 kJ/mol, suggesting that the G53D mutation in histone H2B significantly reduces the interaction between the H2A-H2B dimer and DNA.

The reduced interaction between nucleosomal DNA and histone would potentially affect the biological activities on the chromatin including DNA replication, DNA damage repair, and transcription. To test the effect of H2BG53D on these processes and to decipher the clinical relevance of H2BG53D in PDAC, we employed CRISPR-Cas9 to generate H2BG53D knockin cells in the pancreatic cancer cell line S2VP10 (Supplementary Figs. S4 and S5), wherein we confirmed that this cell line does not harbor the G53D mutation (Table S1). We chose to use a PDAC cell line instead of normal pancreatic cells because we reasoned that the cancer promoting effect of H2BG53D emerges at late stage and would not appear unless in the presence of *KRAS*^G12D^ and under defective cell cycle regulation. The *HIST1H2BO* gene locus was selected for CRISPR/Cas9 targeting for two reasons. (1) The HIST1H2BO was originally found mutated in two PDAC patient samples (Fig. 1a). (2) Among the H2B genes that were found with the H2BG53D mutation, single-guide RNAs targeting this *HIST1H2BO* locus has the highest specificity scores. We tagged both the wild-type and G53D-H2B with FLAG at the C-terminus for further analyses as we reasoned that antibodies specific to the G53D-H2B might not detect the mutant H2B in the context of an assembled nucleosome. Individual clones were selected and genotyped to confirm the accuracy of gene targeting (Fig. S5). The top 20 predicted off-targeting sites were examined by Sanger sequencing, revealing that unspecific gene editing was minimal in our CRISPR clones (Table S2). The knockin of H2BG53D mutation in S2VP10 neither affect the levels of the tested histone modifications (Fig. S6a) nor the loading of the H2B to the chromatin (Supplementary Fig. S6b).

We tested whether the H2BG53D mutation affects DNA damage repair and DNA replication by treating our CRISPR/Cas9 knockin cells with DNA damage agent CPT (topoisomerase 1 inhibitor) and MMS (DNA alkylating agent, generates DNA damage). Using gamma H2AX and 53BP1 as markers of DNA damage, we found that the H2BG53D knockin lines did not exhibit increased sensitivity when compared to the isogenic wild-type knockin clones (Supplementary Fig. S7a, b). In addition, bromodeoxyuridine (BrdU) incorporation assay showed that the mutant lines had no defect in DNA replication (Supplementary Fig. S7c), suggesting that the H2BG53D mutation might not alter DNA damage repair and DNA replication in mammalian cells. To explore the effect of the H2BG53D mutation on transcription directly, we performed in vitro transcription elongation assay using mammalian RNA polymerase II (Pol II) (Fig. 1h). In both the wild-type and H2BG53D nucleosomes, the majority of Pol II stopped at +15, +25, and +45 regions of the nucleosome at 40 mM salt condition. As the salt concentration increased, the Pol II’s nucleosomal passage efficiencies increased in both the wild-type and H2BG53D nucleosomes. We observed that significantly more Pol II passed through the H2BG53D nucleosome than the wild-type nucleosome (Fig. 1i, j). Note that the passage increase by the single mutation was similar to histone tail acetylation, which is well known as a marker for active transcription.^10^ Finally, to elucidate the effect of H2BG53D in cancer development, we performed oncogenic assays and found that the H2BG53D mutation did not affect cell proliferation (Supplementary Fig. S8a). However, the H2BG53D cells displayed increased gap closure (Supplementary Fig. S8b) and transwell migration (Supplementary Fig. S9) properties compared to the isogenic wild-type clones.

In summary, we identified the H2BG53D as a novel cancer-promoting histone mutation and uncovered its effect on enhancing transcription elongation possibly via weakening the interaction between nucleosomal DNA and the histone octamer. These findings, together with the cancer phenotype-promoting effect shown in our CRISPR-Cas9 knockin cells, provided insights into understanding the significance of H2BG53D in gene regulation and PDAC development.

## Supplementary information

Wan et al Supplementary info
