# Supplementary material for: Cancer-associated histone mutation H2BG53D disrupts DNA–histone octamer interaction and promotes oncogenic phenotypes
Source: Signal Transduct Target Ther. 2020 Mar 6;5:27. doi: 10.1038/s41392-020-0131-0 (PMC7060176; doi:10.1038/s41392-020-0131-0)
Supplement: Supplementary file 1 — Wan et al Supplementary info [file 41392_2020_131_MOESM1_ESM.pdf]

Supplementary data - figures and tables

Cancer associated histone mutation H2BG53D disrupts DNA-histone octamer interaction and promotes oncogenic phenotypes

This supplementary data file contains:

- Supplementary Figure 1-9
- Supplementary Table 1-6
- Supplementary Methods
- References

Supplementary Fig.1

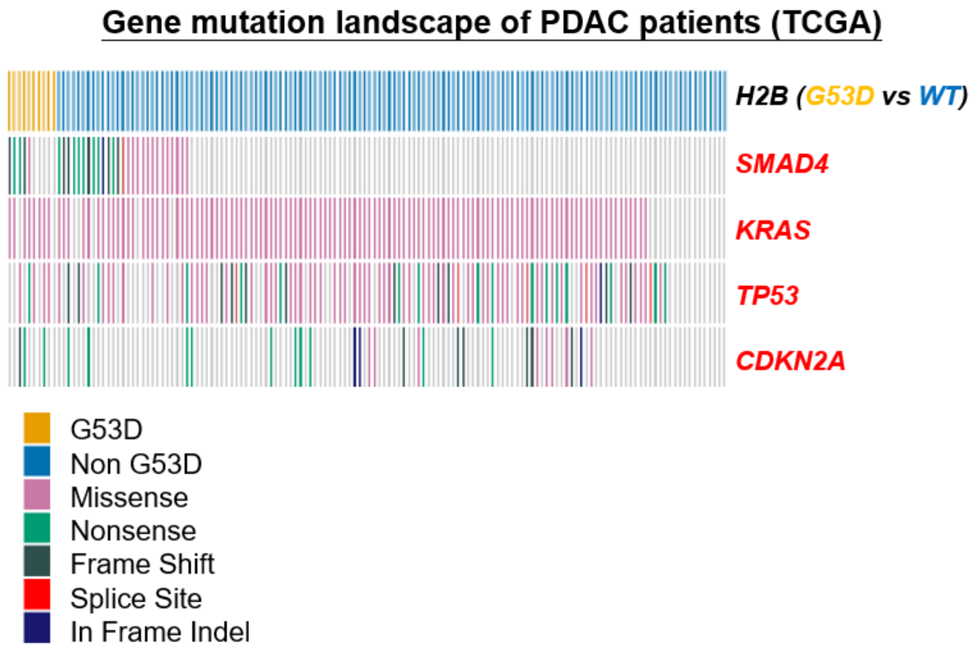

Supplementary Fig.1 | Mutational landscape of PDAC. H2BG53D mutation was found in 10 out of 146 PDAC patients. *KRAS*, *TP53*, *CDKN2A* and *SMAD4* are the four signature driver mutations of pancreatic ductal adenocarinoma. Data was downloaded from TCGA.

Supplementary Fig.2

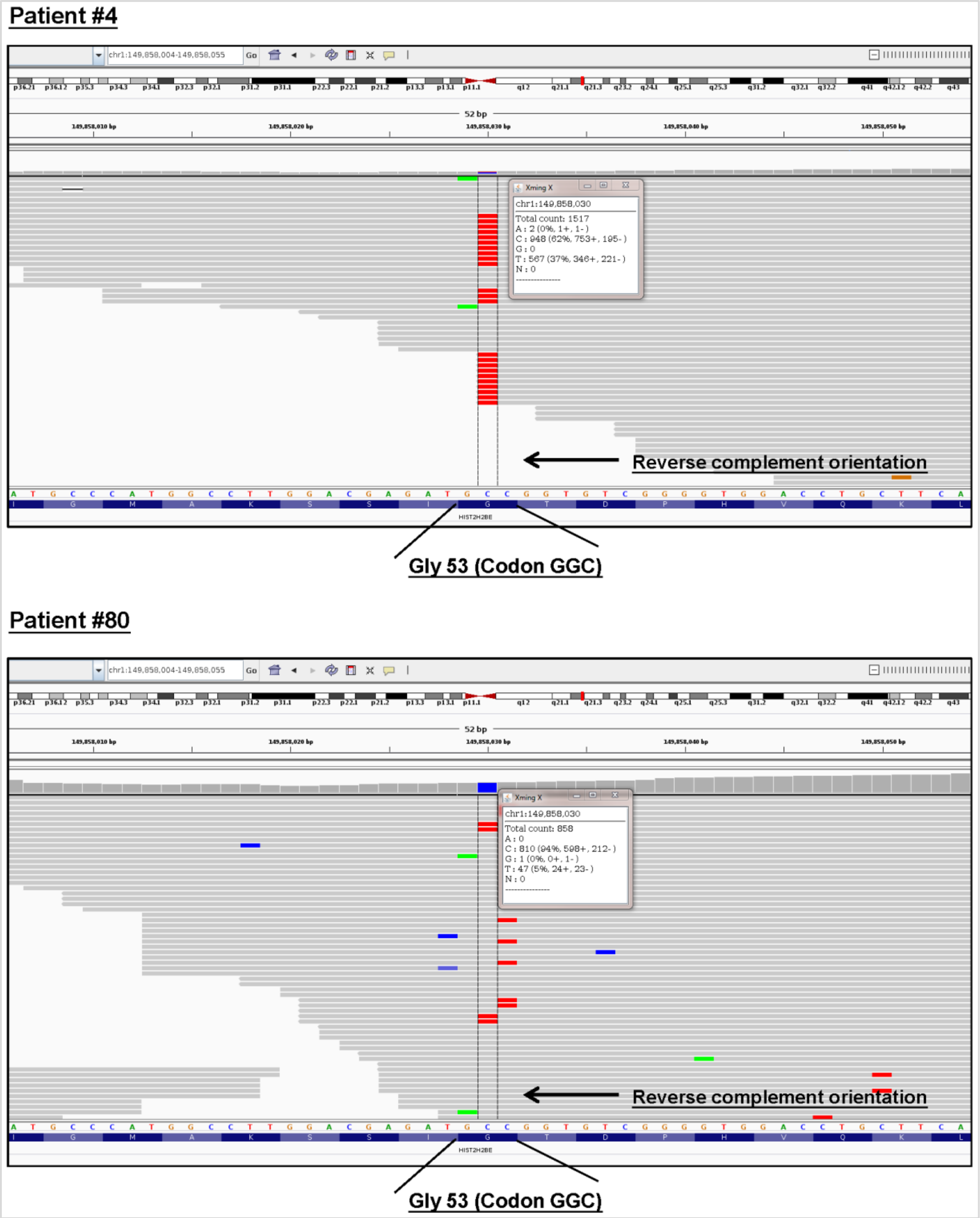

Supplementary Fig.2 | H2B targeted sequencing. H2BG53D mutation was found on *HIST2H2BE* of patient#4 and patient#80. Allele frequencies of the G53D allele in patients #4 and #80 are 37.33% and 5.47% respectively.

Supplementary Fig.3

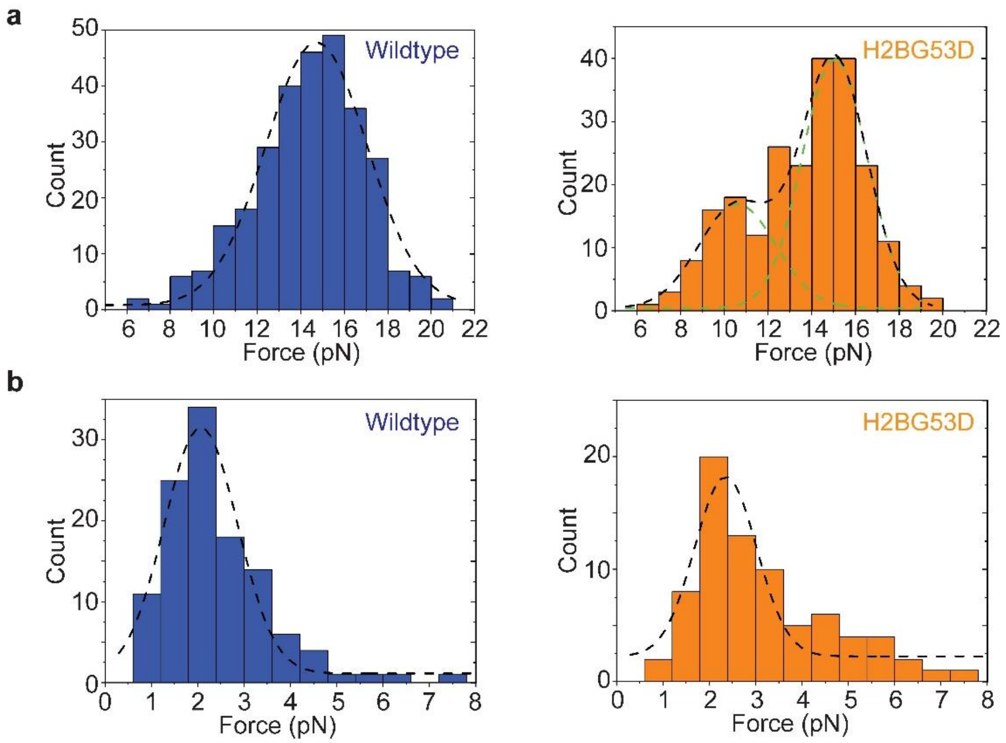

c

	Unwrap			Rewrap		
	Force (pN)	Mean rip size (nm)	N	Force (pN)	Mean rip size (nm)	N
**Wildtype**	14.93±0.32	24.89±1.57	293	2.08±0.07	22.43±3.28	119
**H2BG53D**	15.04±0.27	24.92±1.64	227	2.31±0.10	21.15±2.76	90

d

	N (Unwrap/Rewrap)	Mean rip size (nm)
**Wildtype**	490/435	21.68±2.18
**H2BG53D**	179/139	20.98±2.22

Supplementary Fig.3 | G53D mutant alters histone/DNA interaction (a) The inner rip unwrapping **(b)** or rewrapping forces histograms of the canonical (blue) and the H2BG53D (orange) nucleosomes. The black line represents the Gaussian fit of disruption of histone-DNA interaction at the inner rip and the green line represents the second Gaussian fit of the partial histone-DNA interaction. **(c)** Table summarizing the inner unwrap and rewrap from the nucleosome pulling experiment. **(d)** Table summarizing the outer unwrap and rewrap from the nucleosome hopping experiment.

Supplementary Fig.4

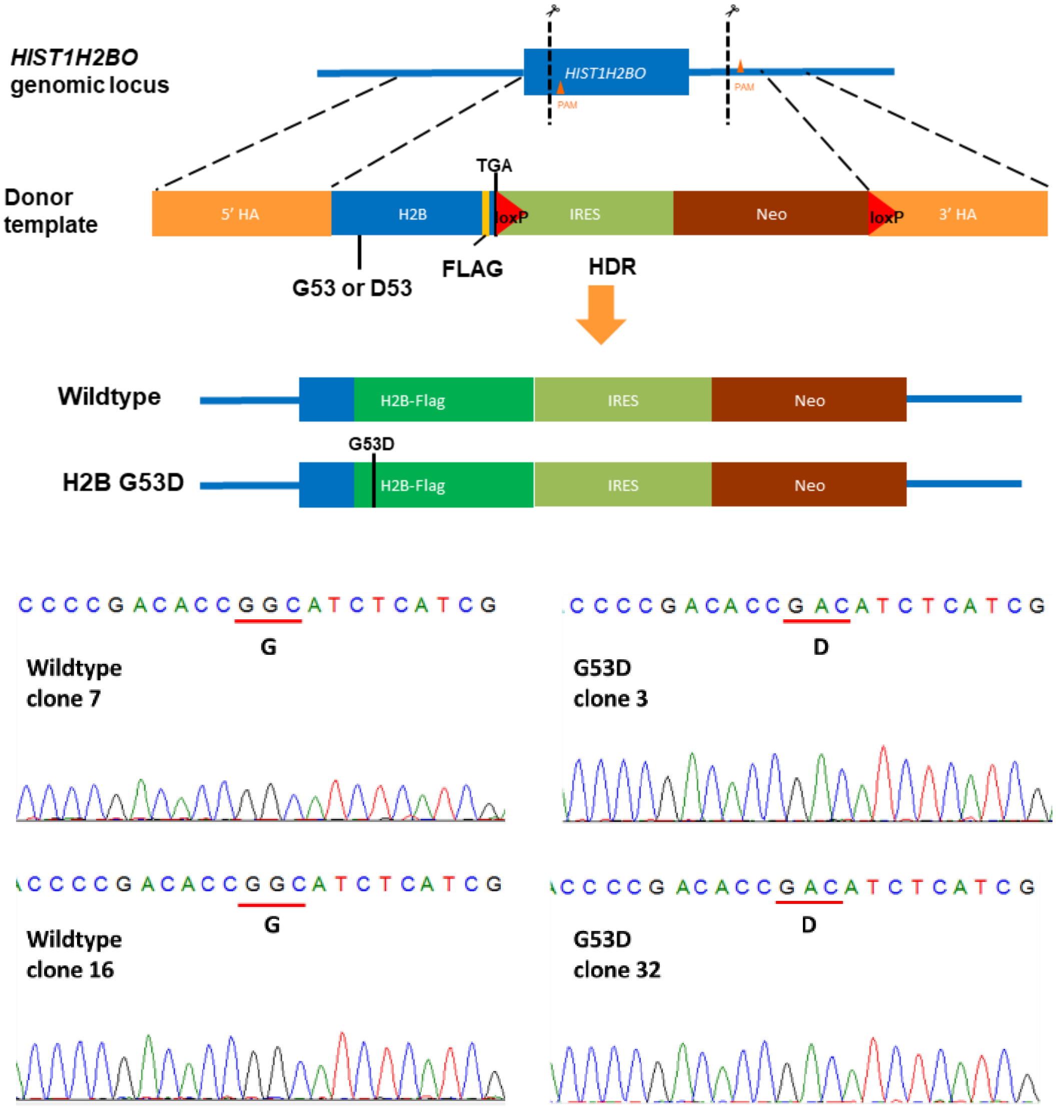

Supplementary Fig.4 | CRISPR/Cas9-mediated gene editing of the *HIST1H2BO* locus in S2VP10. Chromatogram showing Sanger sequencing result from primer pair 2, red line highlighting the codon G53/D53.

Supplementary Fig.5

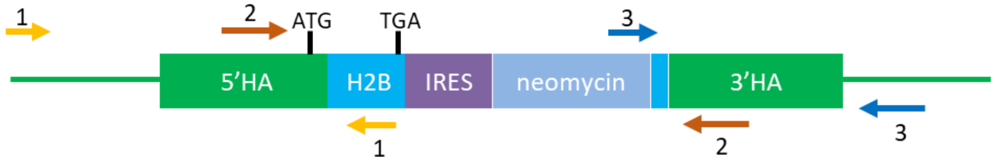

	Purpose	Product size
Pair 1	Checking for the correct integration on the 5' side	1500bp
Pair 2	Checking for the presence of the donor template	700bp (parental allele)<br>2000bp (knock-in allele)
Pair 3	Checking for the correct integration on the 3' side	1200bp

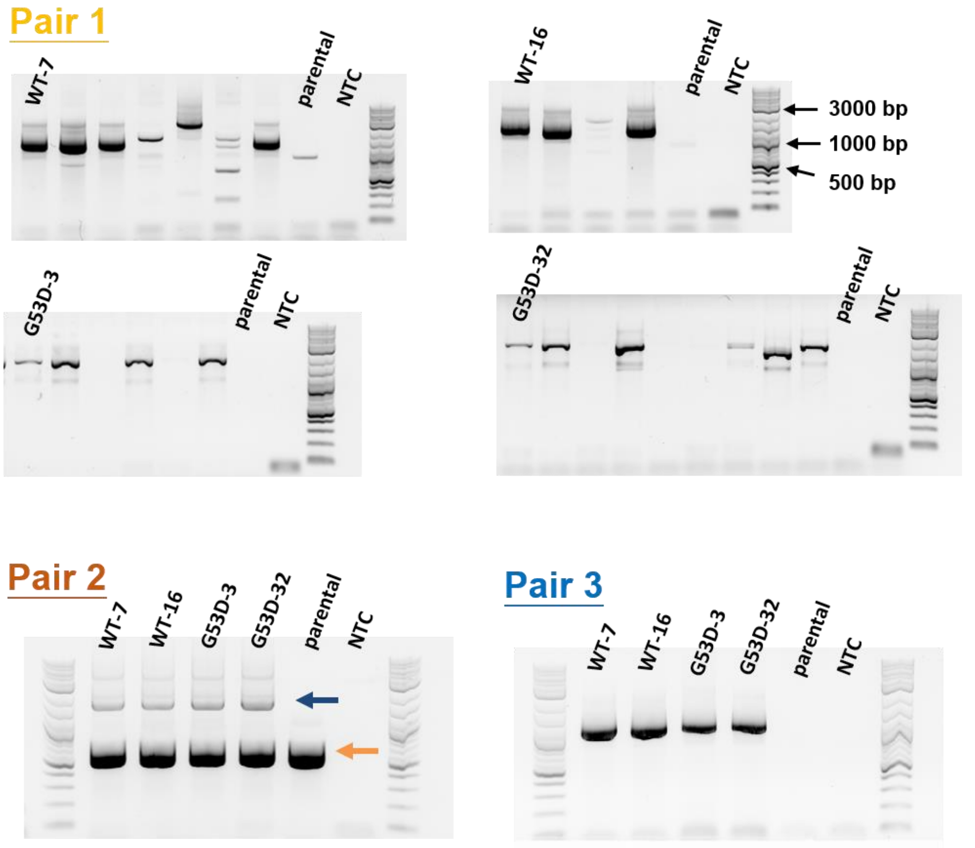

Supplementary Fig.5 | Genotyping strategies for CRISPR-Cas9 generated cell lines. a) Schematic indicating positions of primers used for genotyping. **b)** Genotyping with primer pair 2 showed that both wildtype allele (orange arrow) and knock-in allele (blue arrow) are present in the knock-in clones.

Supplementary Fig.6

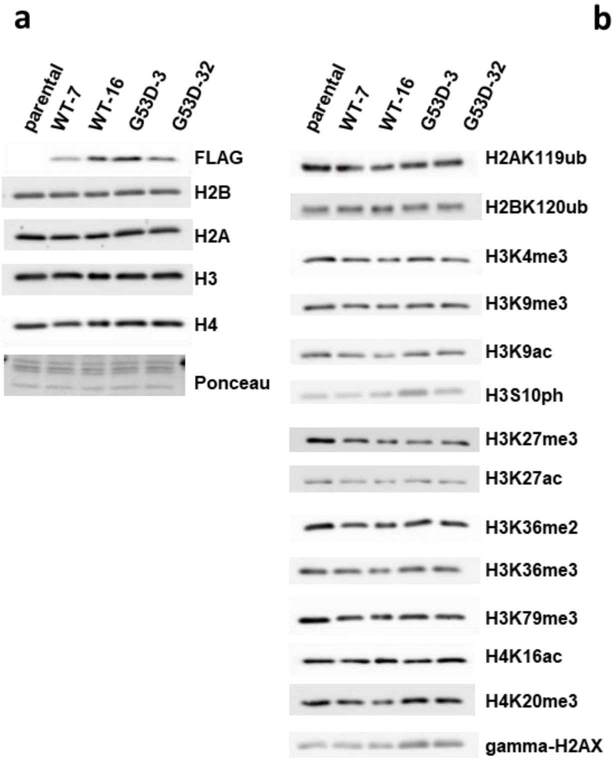

Supplementary Fig.6 | Characterization of CRISPR-Cas9 generated G53D cell lines. (a) Total nuclear extract was collected from the parental S2VP10 cells, two wildtype clones and two H2BG53D knock-in clones were subjected to Western Blot. Levels of histone modifications were determined using antibodies specific to the indicated histone modifications. **(b)** S2VP10 and the isogenic CRISPR knock-in lines were first lysed with hypotonic buffer to obtain the S2 fraction (cytoplasmic, tubulin as control), the pellet was then fractionated into S3 (soluble chromatin) and P3 (insoluble chromatin, H2B and H4 as controls).

Supplementary Fig.7

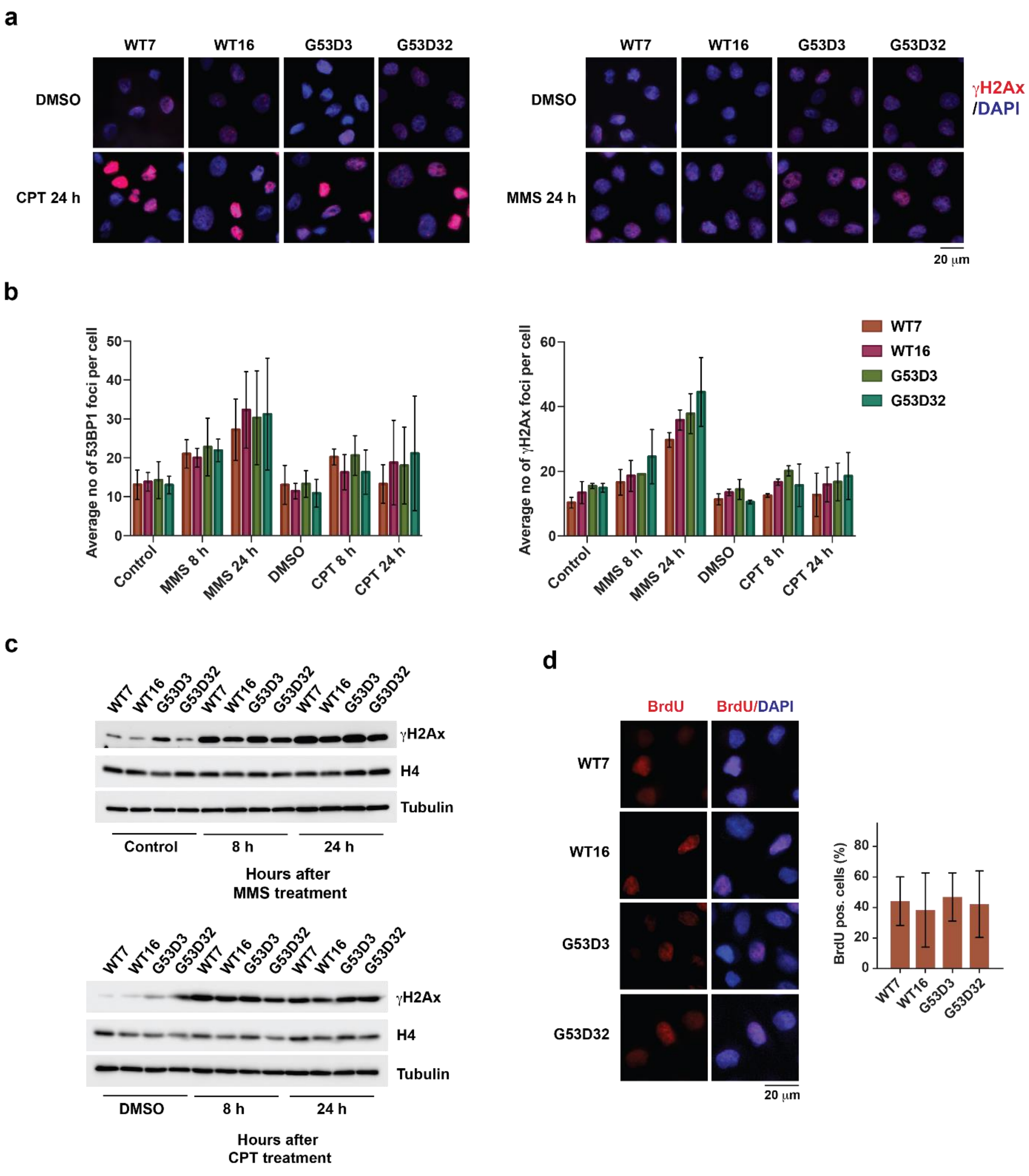

Supplementary Fig.7 | H2BG53D affects DNA damage repair in yeast cells but not in human cells. **(a)** Representative images showing 53BP1 and gH2Ax foci after CPT/MMS treatment. **(b)** Quantification of the average number of 53BP1 and gH2Ax foci per cell. **(c)** Immunoblots of whole cell lysates showing γH2Ax levels. **(d)** BrdU incorporation was analyzed by immunofluorescence staining.

Supplementary Fig.8

a

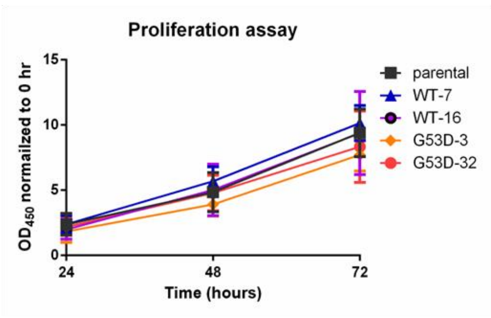

b

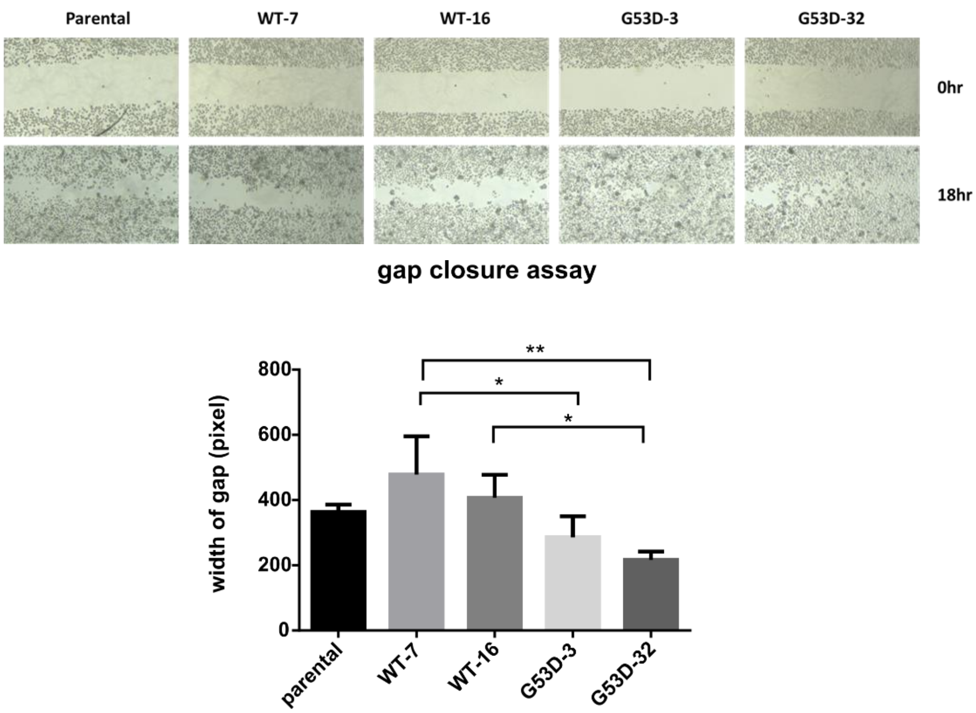

Supplementary Fig.8 | H2BG53D cells acquired cell migration property (a) CCK-8 cell proliferation assay. All measurements were done in triplicate (technical) and normalized to OD_{450} at 0 hour. Error bar showing standard deviation from 3 independent experiments. **(b)** gap closure assay. All samples were done in duplicates, figure showing representative images of 3 independent experiments.

Supplementary Fig.9

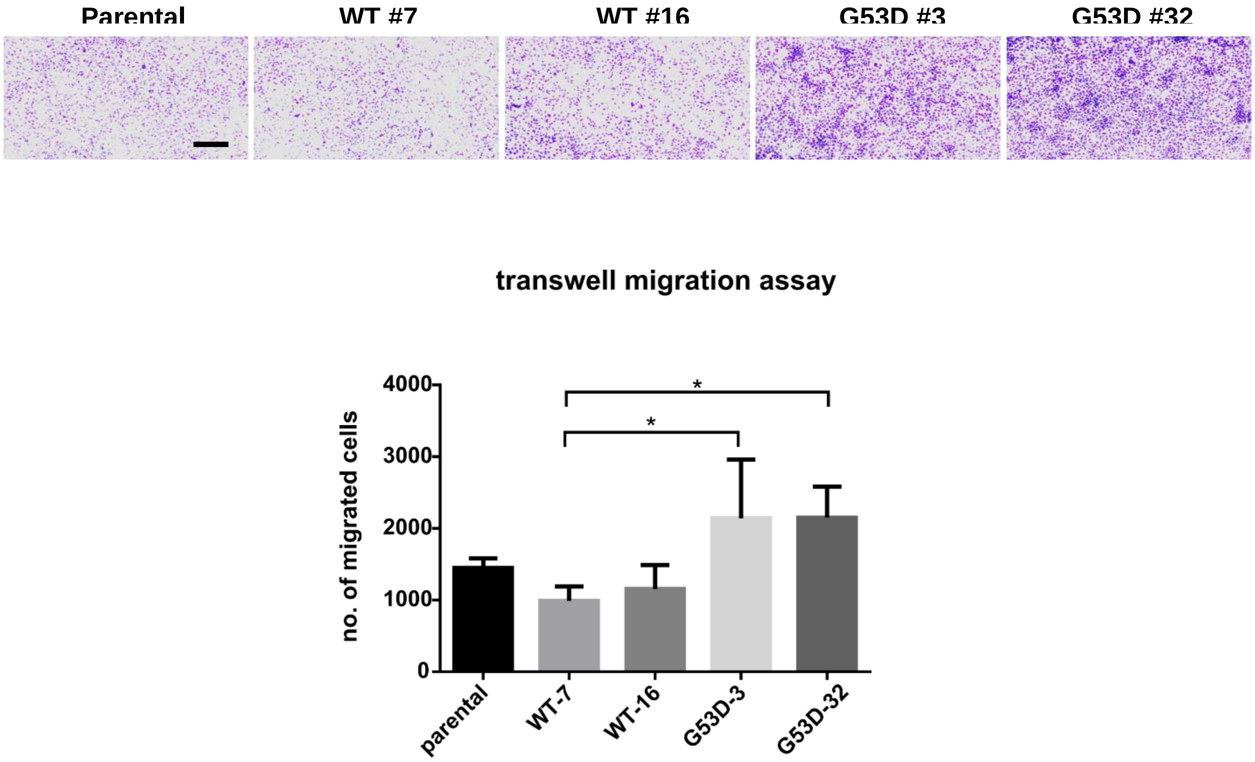

Supplementary Fig.9 | H2BG53D cells acquired transwell migration property (a) Transwell migration assay. All samples were done in duplicates, figure showing representative images of 3 independent experiments. Scale bar, 200µm

Supplementary Table 1

Gene ID	Gene	expected codon usage (Glycine)	sanger sequencing result
**3018**	HSIT1H2BB	GGC	GGC
**8347**	HIST1H2BC	GGC	GGC
**3017**	HIST1H2BD	GGC	GGC
**8344**	HIST1H2BE	GGC	GGC
**8343**	HIST1H2BF	GGC	GGC
**8339**	HIST1H2BG	GGC	GGC
**8345**	HIST1H2BH	GGC	GGC
**8346**	HIST1H2BI	GGC	GGC
**8970**	HIST1H2BJ	GGC	GGC
**85236**	HSIT1H2BK	GGC	GGC
**8340**	HIST1H2BL	GGC	GGC
**8342**	HSIT1H2BM	GGC	GGC
**8341**	HSIT1H2BN	GGT	GGT
**8348**	HIST1H2BO	GGC	GGC
**8349**	HIST2H2BE	GGC	GGC
**440689**	HIST2H2BF	GGC	GGC
**128312**	HIST3H2BB	GGC	GGC

Supplementary Table 1 | Targeted Sanger sequencing of all the H2B genes in S2VP10. Sanger sequencing results confirmed the presence of glycine at position 53 in all the 17 genes encoding histone H2B.

Supplementary Table 2

Target site	Target sequence (mismatch in red)	# mismathes	On target score	Chr	Strand	Position	Sequencing results of Parental/WT7/WT16/G53D3/G53D32
5' gRNA target site	GGAGCAGATTTAGCCGGGTC	0	100	chr6	-1	27861243	
5' off-target site 1	AGAGCAGGTACAGCCGGGTC	4	0.743261097	chrY	1	13310607	no off-target event identified compared to Parental
5' off-target site 2	TGAGCAGACTTAGCAGGGTC	3	0.515955556	chr12	-1	123983633	no off-target event identified compared to Parental
5' off-target site 3	GGAGGAGACAGAGCCGGGTC	4	0.426292988	chr15	1	59447921	no off-target event identified compared to Parental
5' off-target site 4	GGGACAGACTTGGCCGGGTC	4	0.424071175	chr1	1	161369988	no off-target event identified compared to Parental
5' off-target site 5	GGAACAGATTTATCCGGGGC	3	0.395930769	chr17	1	18554732	no off-target event identified compared to Parental
5' off-target site 6	GGAACAGATTTATCCGGGGC	3	0.395930769	chr5	-1	43529893	no off-target event identified compared to Parental
5' off-target site 7	ACAGAAGATTTAGCCGGATC	4	0.321774194	chr1	1	5495269	no off-target event identified compared to Parental
5' off-target site 8	GGGGTAGATTCAGCCGGGCC	4	0.277870889	chr11	-1	125074688	no off-target event identified compared to Parental
5' off-target site 9	CCAGCAGAATTAGCTGGGTC	4	0.254738974	chr18	-1	66309528	no off-target event identified compared to Parental
5' off-target site 10	TTAGCAGATTAAGCAGGGTC	4	0.231391376	chr10	-1	112255820	no off-target event identified compared to Parental
3' gRNA target site	TCTTTTGGGTGGACTCCGGC	0	100	chr6	1	27861705	
3' off-target site 1	TGTCTTGGGTGGACTCTGGC	3	1.25042735	chr1	1	227173781	no off-target event identified compared to Parental
3' off-target site 2	TCTTGTGTGTGGACTCCTGC	3	0.599677939	chr17	-1	27222984	no off-target event identified compared to Parental
3' off-target site 3	TCTTTTGGGAGGACTCTGGG	3	0.416203993	chr11	1	63763805	no off-target event identified compared to Parental
3' off-target site 4	TCTATTCTGTGGACTCTGGC	4	0.402050295	chr4	1	129149438	no off-target event identified compared to Parental
3' off-target site 5	ACTTTTGCTTGGACTCGGGC	4	0.379196691	chr1	1	5041357	no off-target event identified compared to Parental
3' off-target site 6	CCTGTGGGGTGGACTCTGGC	4	0.375472992	chr9	1	34363031	no off-target event identified compared to Parental
3' off-target site 7	TCTTGTGGGTGGACCCCGGA	3	0.362968205	chr7	1	102158002	no off-target event identified compared to Parental
3' off-target site 8	TCTTGTGGGTGGACCCCGGA	3	0.362968205	chr7	1	102256981	no off-target event identified compared to Parental
3' off-target site 9	TCTTGTGTCTGGACTCTGGC	4	0.353596915	chr10	1	82259029	no off-target event identified compared to Parental
3' off-target site 10	TCTTTTGCTAGGACTCCGGG	4	0.352730832	chr5	-1	1239117	no off-target event identified compared to Parental

Supplementary Table 2 | Off-targeted gene editing is minimal in S2VP10 CRISPR knock-in cell line. Potential off-targeting sites for the two sgRNAs used in this work was predicted using http://crispr.mit.edu (discontinued). Nucleotide in red indicates mismatches to the on-target sequence.

Supplementary Table 3

Oligo name	Oligo sequence
**TOP001**	TACACGACGCTCTTCCGATCTNNWNNWNATGCCANTGCCGTT
**TOP002**	TACACGACGCTCTTCCGATCTNNWNNWNACCATCNCAACGAT
**TOP003**	TACACGACGCTCTTCCGATCTNNWNNWNGTGGCCNTACTACT
**TOP004**	TACACGACGCTCTTCCGATCTNNWNNWNGGAGTTNGAGGTGT
**TOP005**	TACACGACGCTCTTCCGATCTNNWNNWNATTGCANAGCAACT
**TOP006**	TACACGACGCTCTTCCGATCTNNWNNWNTACAACNCGAGTAT
**TOP007**	TACACGACGCTCTTCCGATCTNNWNNWNTGCGTTNCTAGCGT
**TOP008**	TACACGACGCTCTTCCGATCTNNWNNWNTGTTCCNTCTCACT
**TOP009**	TACACGACGCTCTTCCGATCTNNWNNWNTGCAGTNCCTCGAT
**TOP010**	TACACGACGCTCTTCCGATCTNNWNNWNCGATACNACTGCCT
**TOP011**	TACACGACGCTCTTCCGATCTNNWNNWNTCTGCGNAGTCTGT
**TOP012**	TACACGACGCTCTTCCGATCTNNWNNWNTCTTGCNGGAGTCT
**TOP013**	TACACGACGCTCTTCCGATCTNNWNNWNAGGCTTNACGTGTT
**TOP014**	TACACGACGCTCTTCCGATCTNNWNNWNTCACGANGTCACAT
**TOP015**	TACACGACGCTCTTCCGATCTNNWNNWNAGCCGGNAGAGTAT
**TOP016**	TACACGACGCTCTTCCGATCTNNWNNWNATGGAANGGTGGCT
**TOP017**	TACACGACGCTCTTCCGATCTNNWNNWNAGACACNCAATGTT
**TOP018**	TACACGACGCTCTTCCGATCTNNWNNWNGGTAGTNTCATAGT
**TOP019**	TACACGACGCTCTTCCGATCTNNWNNWNATCGAGNGATCTAT
**TOP020**	TACACGACGCTCTTCCGATCTNNWNNWNCAACAANTGCCAAT
**TOP021**	TACACGACGCTCTTCCGATCTNNWNNWNGTGCAANCCAATCT
**TOP022**	TACACGACGCTCTTCCGATCTNNWNNWNACAATGNTCACAGT
**TOP023**	TACACGACGCTCTTCCGATCTNNWNNWNGGCTCTNAACGTAT
**TOP024**	TACACGACGCTCTTCCGATCTNNWNNWNCTCCACNATTCCTT
**TOP025**	TACACGACGCTCTTCCGATCTNNWNNWNAAGGCGNCTCCTTT
**TOP026**	TACACGACGCTCTTCCGATCTNNWNNWNGAGACANGTGGAAT
**TOP027**	TACACGACGCTCTTCCGATCTNNWNNWNATGCGTNAATGCAT
**TOP028**	TACACGACGCTCTTCCGATCTNNWNNWNTACAAGNTGGTCCT
**TOP029**	TACACGACGCTCTTCCGATCTNNWNNWNACTCCTNTGTGTTT
**TOP030**	TACACGACGCTCTTCCGATCTNNWNNWNTGGAGCNCTTGTCT
**TOP031**	TACACGACGCTCTTCCGATCTNNWNNWNCAACTGNTCAGACT
**TOP032**	TACACGACGCTCTTCCGATCTNNWNNWNTCAGATNACCAGCT
**TOP033**	TACACGACGCTCTTCCGATCTNNWNNWNTGGCCGNTTACTGT
**TOP034**	TACACGACGCTCTTCCGATCTNNWNNWNGGTGCTNAATCACT
**TOP035**	TACACGACGCTCTTCCGATCTNNWNNWNCATACGNATACAGT
**TOP036**	TACACGACGCTCTTCCGATCTNNWNNWNTGAATANCCTGGCT
**TOP037**	TACACGACGCTCTTCCGATCTNNWNNWNGTGGTCNATCGTAT
**TOP038**	TACACGACGCTCTTCCGATCTNNWNNWNGAACCTNATGACAT
**TOP039**	TACACGACGCTCTTCCGATCTNNWNNWNACACGANCTATAGT
**TOP040**	TACACGACGCTCTTCCGATCTNNWNNWNATATGCNGAGACTT

**TOP041**	TACACGACGCTCTTCCGATCTNNWNNWNCGCTTANGTGCTGT
**TOP042**	TACACGACGCTCTTCCGATCTNNWNNWNACTACTNGAGGATT
**TOP043**	TACACGACGCTCTTCCGATCTNNWNNWNGCTCCGNACCATAT
**TOP044**	TACACGACGCTCTTCCGATCTNNWNNWNTTCGGCNATAGTGT
**TOP045**	TACACGACGCTCTTCCGATCTNNWNNWNTTAGAGNCCATGCT
**TOP046**	TACACGACGCTCTTCCGATCTNNWNNWNAGGTGANGTTCTAT
**TOP047**	TACACGACGCTCTTCCGATCTNNWNNWNAACATTNGCAGGTT
**TOP048**	TACACGACGCTCTTCCGATCTNNWNNWNGGTGGCNATGGAAT
**TOP049**	TACACGACGCTCTTCCGATCTNNWNNWNGACCGTNCAATACT
**TOP050**	TACACGACGCTCTTCCGATCTNNWNNWNCTGTATNGGAGCTT
**TOP051**	TACACGACGCTCTTCCGATCTNNWNNWNATTAGCNAGCGTAT
**TOP052**	TACACGACGCTCTTCCGATCTNNWNNWNCCGTATNATGCGCT
**TOP053**	TACACGACGCTCTTCCGATCTNNWNNWNGGTTCGNGTCCATT
**TOP054**	TACACGACGCTCTTCCGATCTNNWNNWNCCAATCNGTGCAAT
**TOP055**	TACACGACGCTCTTCCGATCTNNWNNWNGGTTAGNAGCGGAT
**TOP056**	TACACGACGCTCTTCCGATCTNNWNNWNAGAGCANTCCACTT
**TOP057**	TACACGACGCTCTTCCGATCTNNWNNWNACCGAANCAATCCT
**TOP058**	TACACGACGCTCTTCCGATCTNNWNNWNGTTCTANAGGTGAT
**TOP059**	TACACGACGCTCTTCCGATCTNNWNNWNCTACAANTTGAGGT
**TOP060**	TACACGACGCTCTTCCGATCTNNWNNWNTCGAAGNACGTATT
**TOP061**	TACACGACGCTCTTCCGATCTNNWNNWNCCAATANCGCCTGT
**TOP062**	TACACGACGCTCTTCCGATCTNNWNNWNCGGTCANATTGACT
**TOP063**	TACACGACGCTCTTCCGATCTNNWNNWNGCTGCGNTATACCT
**TOP064**	TACACGACGCTCTTCCGATCTNNWNNWNCTGATTNACGAGAT
**TOP065**	TACACGACGCTCTTCCGATCTNNWNNWNATGGCANATTCAGT
**TOP066**	TACACGACGCTCTTCCGATCTNNWNNWNACTGTANCATGAGT
**TOP067**	TACACGACGCTCTTCCGATCTNNWNNWNGCATCGNTCTGGTT
**TOP068**	TACACGACGCTCTTCCGATCTNNWNNWNTCGCGTNCCAGTAT
**TOP069**	TACACGACGCTCTTCCGATCTNNWNNWNAGCGTANATTAGCT
**TOP070**	TACACGACGCTCTTCCGATCTNNWNNWNCTTGACNGAGGTTT
**TOP071**	TACACGACGCTCTTCCGATCTNNWNNWNGCTGGTNCTAGTCT
**TOP072**	TACACGACGCTCTTCCGATCTNNWNNWNGACCTGNAATACAT
**TOP073**	TACACGACGCTCTTCCGATCTNNWNNWNCAGATTNAACCAGT
**TOP074**	TACACGACGCTCTTCCGATCTNNWNNWNATCACANTTCTCCT
**TOP075**	TACACGACGCTCTTCCGATCTNNWNNWNAGTATTNCGCGCAT
**TOP076**	TACACGACGCTCTTCCGATCTNNWNNWNGGCTTANCTTGGAT
**TOP077**	TACACGACGCTCTTCCGATCTNNWNNWNCTCGGTNCAACCAT
**TOP078**	TACACGACGCTCTTCCGATCTNNWNNWNAAGCACNGTCTCAT
**TOP079**	TACACGACGCTCTTCCGATCTNNWNNWNACCTCTNATTCGTT
**TOP080**	TACACGACGCTCTTCCGATCTNNWNNWNGCCATTNATAGAGT
**TOP081**	TACACGACGCTCTTCCGATCTNNWNNWNACCTTANCACCTTT
**TOP082**	TACACGACGCTCTTCCGATCTNNWNNWNGTGCATNTCGCCAT
**TOP083**	TACACGACGCTCTTCCGATCTNNWNNWNTAGTTCNGGTGACT

**TOP084**	TACACGACGCTCTTCCGATCTNNWNNWNCCTTATNAGAAGGT
**TOP085**	TACACGACGCTCTTCCGATCTNNWNNWNCTTCGCNGGATGTT
**TOP086**	TACACGACGCTCTTCCGATCTNNWNNWNTTCGCTNAACCTTT
**TOP087**	TACACGACGCTCTTCCGATCTNNWNNWNGACTCCNTAGACCT
**TOP088**	TACACGACGCTCTTCCGATCTNNWNNWNTCGGTANGCAACTT
**TOP089**	TACACGACGCTCTTCCGATCTNNWNNWNAGTTGANGGCATTT
**TOP090**	TACACGACGCTCTTCCGATCTNNWNNWNAAGACGNTAGCGGT
**TOP091**	TACACGACGCTCTTCCGATCTNNWNNWNGGACTCNAACTAAT
**TOP092**	TACACGACGCTCTTCCGATCTNNWNNWNGCAGAGNAGGCTAT
**TOP093**	TACACGACGCTCTTCCGATCTNNWNNWNCATACANCGCACCT
**TOP094**	TACACGACGCTCTTCCGATCTNNWNNWNATGCTCNTAGAGAT
**TOP095**	TACACGACGCTCTTCCGATCTNNWNNWNTGGCATNGTTGGTT
**TOP096**	TACACGACGCTCTTCCGATCTNNWNNWNTGCTCGNATGTGCT
**BOT001**	/5Phos/ACGGCANTGGCAT/3Phos/
**BOT002**	/5Phos/TCGTTGNGATGGT/3Phos/
**BOT003**	/5Phos/GTAGTANGGCCAC/3Phos/
**BOT004**	/5Phos/CACCTCNAACTCC/3Phos/
**BOT005**	/5Phos/GTTGCTNTGCAAT/3Phos/
**BOT006**	/5Phos/TACTCGNGTTGTA/3Phos/
**BOT007**	/5Phos/CGCTAGNAACGCA/3Phos/
**BOT008**	/5Phos/GTGAGANGGAACA/3Phos/
**BOT009**	/5Phos/TCGAGGNACTGCA/3Phos/
**BOT010**	/5Phos/GGCAGTNGTATCG/3Phos/
**BOT011**	/5Phos/CAGACTNCGCAGA/3Phos/
**BOT012**	/5Phos/GACTCCNGCAAGA/3Phos/
**BOT013**	/5Phos/ACACGTNAAGCCT/3Phos/
**BOT014**	/5Phos/TGTGACNTCGTGA/3Phos/
**BOT015**	/5Phos/TACTCTNCCGGCT/3Phos/
**BOT016**	/5Phos/GCCACCNTTCCAT/3Phos/
**BOT017**	/5Phos/ACATTGNGTGTCT/3Phos/
**BOT018**	/5Phos/CTATGANACTACC/3Phos/
**BOT019**	/5Phos/TAGATCNCTCGAT/3Phos/
**BOT020**	/5Phos/TTGGCANTTGTTG/3Phos/
**BOT021**	/5Phos/GATTGGNTTGCAC/3Phos/
**BOT022**	/5Phos/CTGTGANCATTGT/3Phos/
**BOT023**	/5Phos/TACGTTNAGAGCC/3Phos/
**BOT024**	/5Phos/AGGAATNGTGGAG/3Phos/
**BOT025**	/5Phos/AAGGAGNCGCCTT/3Phos/
**BOT026**	/5Phos/TTCCACNTGTCTC/3Phos/
**BOT027**	/5Phos/TGCATTNACGCAT/3Phos/
**BOT028**	/5Phos/GGACCANCTTGTA/3Phos/
**BOT029**	/5Phos/AACACANAGGAGT/3Phos/
**BOT030**	/5Phos/GACAAGNGCTCCA/3Phos/

**BOT031**	/5Phos/GTCTGANCAGTTG/3Phos/
**BOT032**	/5Phos/GCTGGTNATCTGA/3Phos/
**BOT033**	/5Phos/CAGTAANCGGCCA/3Phos/
**BOT034**	/5Phos/GTGATTNAGCACC/3Phos/
**BOT035**	/5Phos/CTGTATNCGTATG/3Phos/
**BOT036**	/5Phos/GCCAGGNTATTCA/3Phos/
**BOT037**	/5Phos/TACGATNGACCAC/3Phos/
**BOT038**	/5Phos/TGTCATNAGGTTC/3Phos/
**BOT039**	/5Phos/CTATAGNTCGTGT/3Phos/
**BOT040**	/5Phos/AGTCTCNGCATAT/3Phos/
**BOT041**	/5Phos/CAGCACNTAAGCG/3Phos/
**BOT042**	/5Phos/ATCCTCNAGTAGT/3Phos/
**BOT043**	/5Phos/TATGGTNCGGAGC/3Phos/
**BOT044**	/5Phos/CACTATNGCCGAA/3Phos/
**BOT045**	/5Phos/GCATGGNCTCTAA/3Phos/
**BOT046**	/5Phos/TAGAACNTCACCT/3Phos/
**BOT047**	/5Phos/ACCTGCNAATGTT/3Phos/
**BOT048**	/5Phos/TTCCATNGCCACC/3Phos/
**BOT049**	/5Phos/GTATTGNACGGTC/3Phos/
**BOT050**	/5Phos/AGCTCCNATACAG/3Phos/
**BOT051**	/5Phos/TACGCTNGCTAAT/3Phos/
**BOT052**	/5Phos/GCGCATNATACGG/3Phos/
**BOT053**	/5Phos/ATGGACNCGAACC/3Phos/
**BOT054**	/5Phos/TTGCACNGATTGG/3Phos/
**BOT055**	/5Phos/TCCGCTNCTAACC/3Phos/
**BOT056**	/5Phos/AGTGGANTGCTCT/3Phos/
**BOT057**	/5Phos/GGATTGNTTCGGT/3Phos/
**BOT058**	/5Phos/TCACCTNTAGAAC/3Phos/
**BOT059**	/5Phos/CCTCAANTTGTAG/3Phos/
**BOT060**	/5Phos/ATACGTNCTTCGA/3Phos/
**BOT061**	/5Phos/CAGGCGNTATTGG/3Phos/
**BOT062**	/5Phos/GTCAATNTGACCG/3Phos/
**BOT063**	/5Phos/GGTATANCGCAGC/3Phos/
**BOT064**	/5Phos/TCTCGTNAATCAG/3Phos/
**BOT065**	/5Phos/CTGAATNTGCCAT/3Phos/
**BOT066**	/5Phos/CTCATGNTACAGT/3Phos/
**BOT067**	/5Phos/ACCAGANCGATGC/3Phos/
**BOT068**	/5Phos/TACTGGNACGCGA/3Phos/
**BOT069**	/5Phos/GCTAATNTACGCT/3Phos/
**BOT070**	/5Phos/AACCTCNGTCAAG/3Phos/
**BOT071**	/5Phos/GACTAGNACCAGC/3Phos/
**BOT072**	/5Phos/TGTATTNCAGGTC/3Phos/
**BOT073**	/5Phos/CTGGTTNAATCTG/3Phos/

**BOT074**	/5Phos/GGAGAANTGTGAT/3Phos/
**BOT075**	/5Phos/TGCGCGNAATACT/3Phos/
**BOT076**	/5Phos/TCCAAGNTAAGCC/3Phos/
**BOT077**	/5Phos/TGGTTGNACCGAG/3Phos/
**BOT078**	/5Phos/TGAGACNGTGCTT/3Phos/
**BOT079**	/5Phos/ACGAATNAGAGGT/3Phos/
**BOT080**	/5Phos/CTCTATNAATGGC/3Phos/
**BOT081**	/5Phos/AAGGTGNTAAGGT/3Phos/
**BOT082**	/5Phos/TGGCGANATGCAC/3Phos/
**BOT083**	/5Phos/GTCACCNGAACTA/3Phos/
**BOT084**	/5Phos/CCTTCTNATAAGG/3Phos/
**BOT085**	/5Phos/ACATCCNGCGAAG/3Phos/
**BOT086**	/5Phos/AAGGTTNAGCGAA/3Phos/
**BOT087**	/5Phos/GGTCTANGGAGTC/3Phos/
**BOT088**	/5Phos/AGTTGCNTACCGA/3Phos/
**BOT089**	/5Phos/AATGCCNTCAACT/3Phos/
**BOT090**	/5Phos/CCGCTANCGTCTT/3Phos/
**BOT091**	/5Phos/TTAGTTNGAGTCC/3Phos/
**BOT092**	/5Phos/TAGCCTNCTCTGC/3Phos/
**BOT093**	/5Phos/GGTGCGNTGTATG/3Phos/
**BOT094**	/5Phos/TCTCTANGAGCAT/3Phos/
**BOT095**	/5Phos/ACCAACNATGCCA/3Phos/
**BOT096**	/5Phos/GCACATNCGAGCA/3Phos/
**P558**	AATGATACGGCGACCACCGAGATCTACACTCTTTCCCTACACGACGCTCTTCCGATCT
**i701**	CAAGCAGAAGACGGCATACGAGATTCGCCTTAGTGACTGGAGTTCAGACGTGT
**i702**	CAAGCAGAAGACGGCATACGAGATCTAGTACGGTGACTGGAGTTCAGACGTGT
**i703**	CAAGCAGAAGACGGCATACGAGATTTCTGCCTGTGACTGGAGTTCAGACGTGT
**i704**	CAAGCAGAAGACGGCATACGAGATGCTCAGGAGTGACTGGAGTTCAGACGTGT
**i705**	CAAGCAGAAGACGGCATACGAGATAGGAGTCCGTGACTGGAGTTCAGACGTGT
**i706**	CAAGCAGAAGACGGCATACGAGATCATGCCTAGTGACTGGAGTTCAGACGTGT
**i707**	CAAGCAGAAGACGGCATACGAGATGTAGAGAGGTGACTGGAGTTCAGACGTGT
**i708**	CAAGCAGAAGACGGCATACGAGATCCTCTCTGGTGACTGGAGTTCAGACGTGT
**i709**	CAAGCAGAAGACGGCATACGAGATAGCGTAGCGTGACTGGAGTTCAGACGTGT
**i710**	CAAGCAGAAGACGGCATACGAGATCAGCCTCGGTGACTGGAGTTCAGACGTGT
**i711**	CAAGCAGAAGACGGCATACGAGATTGCCTCTTGTGACTGGAGTTCAGACGTGT
**i712**	CAAGCAGAAGACGGCATACGAGATTCCTCTACGTGACTGGAGTTCAGACGTGT

Supplementary Table 4

Gene	Oligo ID	GSP1
**HIST1H2BB**	HISTH2B-GSP1-1	GGATCTCGACGCTCTCCCTGAAAAGAGCCTTTGGGTTTGG
**HIST1H2BB**	HISTH2B-GSP1-2	GGATCTCGACGCTCTCCCTGTAGTGGAGAGTGTTAGTAGC
**HIST1H2BB**	HISTH2B-GSP1-3	GGATCTCGACGCTCTCCCTCGCACAGCCGTCTGAATCTC
**HIST1H2BB**	HISTH2B-GSP1-4	GGATCTCGACGCTCTCCCTAAGGATGGTAAGAAGCGTAAG
**HIST1H2BB**	HISTH2B-GSP1-5	GGATCTCGACGCTCTCCCTATGGCCTTGGATGAGATGCC
**HIST1H2BB**	HISTH2B-GSP1-6	GGATCTCGACGCTCTCCCTCATCCAAGGCCATGGGGATC
**HIST1H2BC**	HISTH2B-GSP1-7	GGATCTCGACGCTCTCCCTTTGGGGTTAGGTGTTAAGACG
**HIST1H2BC**	HISTH2B-GSP1-8	GGATCTCGACGCTCTCCCTTACTCTCCATCCCTCATTAGC
**HIST1H2BC**	HISTH2B-GSP1-9	GGATCTCGACGCTCTCCCTGCTTACTTGGAATGTTTACTTGG
**HIST1H2BC**	HISTH2B-GSP1-10	GGATCTCGACGCTCTCCCTGACCAAAGCGCAGAAGAAAG
**HIST1H2BC**	HISTH2B-GSP1-11	GGATCTCGACGCTCTCCCTTGGAAGAGATGCCAGTGTCG
**HIST1H2BC**	HISTH2B-GSP1-12	GGATCTCGACGCTCTCCCTCCGACACTGGCATCTCTTCC
**HIST1H2BD**	HISTH2B-GSP1-13	GGATCTCGACGCTCTCCCTGTGTTTGCAACAGTGTTCTAAC
**HIST1H2BD**	HISTH2B-GSP1-14	GGATCTCGACGCTCTCCCTGCCTTTGGGATTAGGTGTAAAG
**HIST1H2BD**	HISTH2B-GSP1-15	GGATCTCGACGCTCTCCCTGAAGAAGGACGGGAAGAAGC
**HIST1H2BD**	HISTH2B-GSP1-16	GGATCTCGACGCTCTCCCTCTTGGCCAGCTCCCCCGGAAG
**HIST1H2BD**	HISTH2B-GSP1-17	GGATCTCGACGCTCTCCCTCAATGGGGATCATGAATTCCTTCG
**HIST1H2BD**	HISTH2B-GSP1-18	GGATCTCGACGCTCTCCCTCTTGGAAGAGATGCCGGTGTC
**HIST1H2BE**	HISTH2B-GSP1-19	GGATCTCGACGCTCTCCCTCCGAGGATCAGGAGATGTAG
**HIST1H2BE**	HISTH2B-GSP1-20	GGATCTCGACGCTCTCCCTGAGCCTTTGGGTTTACTAAGG
**HIST1H2BE**	HISTH2B-GSP1-21	GGATCTCGACGCTCTCCCTCCGTGACCAAGGCGCAGAAG
**HIST1H2BE**	HISTH2B-GSP1-22	GGATCTCGACGCTCTCCCTTTGGCCAGCTCCCCGGGAAG
**HIST1H2BE**	HISTH2B-GSP1-23	GGATCTCGACGCTCTCCCTATCATGAATTCCTTTGTCAACG
**HIST1H2BE**	HISTH2B-GSP1-24	GGATCTCGACGCTCTCCCTATGGCTTTAGAGGAGATGCC
**HIST1H2BF**	HISTH2B-GSP1-25	GGATCTCGACGCTCTCCCTTGCATTTCTCTTTAGGTTGTGG
**HIST1H2BF**	HISTH2B-GSP1-26	GGATCTCGACGCTCTCCCTAAAGAGCCTTTGGGATTGGG
**HIST1H2BF**	HISTH2B-GSP1-27	GGATCTCGACGCTCTCCCTGCAGAAGAAGGATGGTAAGAAGC
**HIST1H2BF**	HISTH2B-GSP1-28	GGATCTCGACGCTCTCCCTGCCTTGGTGCCCTCTGACAC
**HIST1H2BF**	HISTH2B-GSP1-29	GGATCTCGACGCTCTCCCTTGTACGTGTACAAGGTGCTAAAGCAG
**HIST1H2BF**	HISTH2B-GSP1-30	GGATCTCGACGCTCTCCCTCTTGGATGAGATGCCGGTGTC
**HIST1H2BG**	HISTH2B-GSP1-31	GGATCTCGACGCTCTCCCTCGGAAAAATTATTCCCTAGAAAACG
**HIST1H2BG**	HISTH2B-GSP1-32	GGATCTCGACGCTCTCCCTCTATAAAAGGAGAGACTCTAGACAC
**HIST1H2BG**	HISTH2B-GSP1-33	GGATCTCGACGCTCTCCCTGGATCTCCCTGGAGGTAATG
**HIST1H2BG**	HISTH2B-GSP1-34	GGATCTCGACGCTCTCCCTCGCAGAAGAAGGATGGCAAG
**HIST1H2BG**	HISTH2B-GSP1-35	GGATCTCGACGCTCTCCCTATGCCAGTATCGGGGTGAAC
**HIST1H2BG**	HISTH2B-GSP1-36	GGATCTCGACGCTCTCCCTTGGGCATCATGAATTCCTTCG
**HIST1H2BH**	HISTH2B-GSP1-37	GGATCTCGACGCTCTCCCTTACAAAAACGGCCAGCTGTG
**HIST1H2BH**	HISTH2B-GSP1-38	GGATCTCGACGCTCTCCCTCACTTCTCCCATAGAATACAGC
**HIST1H2BH**	HISTH2B-GSP1-39	GGATCTCGACGCTCTCCCTCAAGGCGCAGAAGAAGGATG
**HIST1H2BH**	HISTH2B-GSP1-40	GGATCTCGACGCTCTCCCTACTTGGTGACGGCCTTAGTG

**HIST1H2BH**	HISTH2B-GSP1-41	GGATCTCGACGCTCTCCCTATCATGAATTCCTTTGTCAACG
**HIST1H2BH**	HISTH2B-GSP1-42	GGATCTCGACGCTCTCCCTATGGCTTTGGAGGAGATGCC
**HIST1H2BI**	HISTH2B-GSP1-43	GGATCTCGACGCTCTCCCTTGGTCATTTGACGGTATCAC
**HIST1H2BI**	HISTH2B-GSP1-44	GGATCTCGACGCTCTCCCTCCATTTTACCTTGCCAATGCC
**HIST1H2BI**	HISTH2B-GSP1-45	GGATCTCGACGCTCTCCCTGCTCCAAGAAGGCGGTGACC
**HIST1H2BI**	HISTH2B-GSP1-46	GGATCTCGACGCTCTCCCTGATCTCCCTGGAAGTGATGG
**HIST1H2BI**	HISTH2B-GSP1-47	GGATCTCGACGCTCTCCCTGACATTTTCGAGCGCATTGC
**HIST1H2BI**	HISTH2B-GSP1-48	GGATCTCGACGCTCTCCCTCATAATCCCCATAGCCTTGGACGAG
**HIST1H2BJ**	HISTH2B-GSP1-49	GGATCTCGACGCTCTCCCTTCTTAAAAGAGCCGTTAGGG
**HIST1H2BJ**	HISTH2B-GSP1-50	GGATCTCGACGCTCTCCCTGATTCTATATAAAAGCGCCTTGTC
**HIST1H2BJ**	HISTH2B-GSP1-51	GGATCTCGACGCTCTCCCTTTGGTGACGGCCTTAGTACCC
**HIST1H2BJ**	HISTH2B-GSP1-52	GGATCTCGACGCTCTCCCTCTAAGGCGCAGAAGAAAGACG
**HIST1H2BJ**	HISTH2B-GSP1-53	GGATCTCGACGCTCTCCCTGACGAAATGCCGGTGTCAGG
**HIST1H2BJ**	HISTH2B-GSP1-54	GGATCTCGACGCTCTCCCTATGGCATCATGAATTCGTTTG
**HIST1H2BK**	HISTH2B-GSP1-55	GGATCTCGACGCTCTCCCTGGTTGGGCTTTAAGACGCTTAC
**HIST1H2BK**	HISTH2B-GSP1-56	GGATCTCGACGCTCTCCCTCCCGTTTTCTCGATCTGCTG
**HIST1H2BK**	HISTH2B-GSP1-57	GGATCTCGACGCTCTCCCTCAAGTTTACTTAGCGCTGGTG
**HIST1H2BK**	HISTH2B-GSP1-58	GGATCTCGACGCTCTCCCTCCGTGACTAAGGCGCAGAAG
**HIST1H2BK**	HISTH2B-GSP1-59	GGATCTCGACGCTCTCCCTATGGCCTTAGAGGAGATGCC
**HIST1H2BK**	HISTH2B-GSP1-60	GGATCTCGACGCTCTCCCTCATCTCCTCTAAGGCCATGG
**HIST1H2BL**	HISTH2B-GSP1-61	GGATCTCGACGCTCTCCCTGCCTTTGGGTTGGACAAGAG
**HIST1H2BL**	HISTH2B-GSP1-62	GGATCTCGACGCTCTCCCTAGAGACCTTCCCACTACCTC
**HIST1H2BL**	HISTH2B-GSP1-63	GGATCTCGACGCTCTCCCTGCCAGCTCCCCCGGAAGCAG
**HIST1H2BL**	HISTH2B-GSP1-64	GGATCTCGACGCTCTCCCTAAGAAGGCGGTGACCAAGGC
**HIST1H2BL**	HISTH2B-GSP1-65	GGATCTCGACGCTCTCCCTATGGCCTTAGAAGAGATGCC
**HIST1H2BL**	HISTH2B-GSP1-66	GGATCTCGACGCTCTCCCTGGCATCTCTTCTAAGGCCATG
**HIST1H2BM**	HISTH2B-GSP1-67	GGATCTCGACGCTCTCCCTCATTTAAATAAAGAGGACGAAACAGC
**HIST1H2BM**	HISTH2B-GSP1-68	GGATCTCGACGCTCTCCCTTGTGGGTCACGGCGGAACTG
**HIST1H2BM**	HISTH2B-GSP1-69	GGATCTCGACGCTCTCCCTCGTCTGGATCTCCCTCGAAGTG
**HIST1H2BM**	HISTH2B-GSP1-70	GGATCTCGACGCTCTCCCTCGTCAACGACATCTTTGAGC
**HIST1H2BM**	HISTH2B-GSP1-71	GGATCTCGACGCTCTCCCTATAGCCTTGGAAGAGATGCC
**HIST1H2BN**	HISTH2B-GSP1-72	GGATCTCGACGCTCTCCCTACAGAGCTACCGTCTTCCTG
**HIST1H2BN**	HISTH2B-GSP1-73	GGATCTCGACGCTCTCCCTTGGGCCGAGACTGACCGAAC
**HIST1H2BN**	HISTH2B-GSP1-74	GGATCTCGACGCTCTCCCTTGACAAAGGCCCAGAAGAAGGAC
**HIST1H2BN**	HISTH2B-GSP1-75	GGATCTCGACGCTCTCCCTGGGCTCACTTGGAACTGGTG
**HIST1H2BN**	HISTH2B-GSP1-76	GGATCTCGACGCTCTCCCTACTCCTTCGTCAATGACATC
**HIST1H2BN**	HISTH2B-GSP1-77	GGATCTCGACGCTCTCCCTTGGCCTTGGACGAGATACCG
**HIST1H2BO**	HISTH2B-GSP1-78	GGATCTCGACGCTCTCCCTTAACGTCATCTGAGTAACTTCC
**HIST1H2BO**	HISTH2B-GSP1-79	GGATCTCGACGCTCTCCCTTCCACCCAAAAGACTACAAG
**HIST1H2BO**	HISTH2B-GSP1-80	GGATCTCGACGCTCTCCCTCCAGAAAAAGGACGGCAAGAAG
**HIST1H2BO**	HISTH2B-GSP1-81	GGATCTCGACGCTCTCCCTTCGTCAATGACATCTTTGAGC
**HIST1H2BO**	HISTH2B-GSP1-82	GGATCTCGACGCTCTCCCTCGGTGTCGGGGTGGACTTGC
**HIST2H2BE**	HISTH2B-GSP1-83	GGATCTCGACGCTCTCCCTTGATTAGGTGGGTGGCTCTG

**HIST2H2BE**	HISTH2B-GSP1-84	GGATCTCGACGCTCTCCCTAAGAATCAGGCCCGCCCATTC
**HIST2H2BE**	HISTH2B-GSP1-85	GGATCTCGACGCTCTCCCTCCGTCTGGATCTCGCGGGATG
**HIST2H2BE**	HISTH2B-GSP1-86	GGATCTCGACGCTCTCCCTAGCCGCAAAGAGAGCTACTC
**HIST2H2BE**	HISTH2B-GSP1-87	GGATCTCGACGCTCTCCCTAAAGAGAGCTACTCCATCTACG
**HIST2H2BF**	HISTH2B-GSP1-88	GGATCTCGACGCTCTCCCTTGGGGTTAGGTGGTTGATCTATTGCG
**HIST2H2BF**	HISTH2B-GSP1-89	GGATCTCGACGCTCTCCCTATACGGGCTCTATAAGTAGCG
**HIST2H2BF**	HISTH2B-GSP1-90	GGATCTCGACGCTCTCCCTCCGTCTGGATCTCGCGGGATG
**HIST2H2BF**	HISTH2B-GSP1-91	GGATCTCGACGCTCTCCCTTCCAAAAAGGCTGTTACGAAAGTG
**HIST2H2BF**	HISTH2B-GSP1-92	GGATCTCGACGCTCTCCCTGCTACTCCGTTTACGTGTAC
**HIST3H2BB**	HISTH2B-GSP1-93	GGATCTCGACGCTCTCCCTATCCTGAGCCTTCATTTGAATAC
**HIST3H2BB**	HISTH2B-GSP1-94	GGATCTCGACGCTCTCCCTCTGAAAAGAGCCTTTGGGTTCAGGAC
**HIST3H2BB**	HISTH2B-GSP1-95	GGATCTCGACGCTCTCCCTCCAAGGCACAGAAGAAGGAC
**HIST3H2BB**	HISTH2B-GSP1-96	GGATCTCGACGCTCTCCCTCCTCACTTGGAGCTGGTGTAC
**HIST3H2BB**	HISTH2B-GSP1-97	GGATCTCGACGCTCTCCCTACTCCTTCGTCAATGACATC
**HIST3H2BB**	HISTH2B-GSP1-98	GGATCTCGACGCTCTCCCTTCGAAGATGTCATTGACGAAG

Supplementary Table 5

Gene	Oligo ID	GSP2
**HIST1H2BB**	HISTH2B-GSP2-1	GTGACTGGAGTTCAGACGTGTGCTCTTCCGATCTGAGCCTTTGGGTTTGGAAGTG
**HIST1H2BB**	HISTH2B-GSP2-2	GTGACTGGAGTTCAGACGTGTGCTCTTCCGATCTGGAGAGTGTTAGTAGCTTTTCTATTC
**HIST1H2BB**	HISTH2B-GSP2-3	GTGACTGGAGTTCAGACGTGTGCTCTTCCGATCTGCCGTCTGAATCTCCCTGGAG
**HIST1H2BB**	HISTH2B-GSP2-4	GTGACTGGAGTTCAGACGTGTGCTCTTCCGATCTCGCAAGGAGAGCTATTCTATC
**HIST1H2BB**	HISTH2B-GSP2-5	GTGACTGGAGTTCAGACGTGTGCTCTTCCGATCTGCCTTGGATGAGATGCCGGTGTC
**HIST1H2BB**	HISTH2B-GSP2-6	GTGACTGGAGTTCAGACGTGTGCTCTTCCGATCTTCATGAATTCCTTCGTCAACG
**HIST1H2BC**	HISTH2B-GSP2-7	GTGACTGGAGTTCAGACGTGTGCTCTTCCGATCTGGTGTTAAGACGCTTACTTGG
**HIST1H2BC**	HISTH2B-GSP2-8	GTGACTGGAGTTCAGACGTGTGCTCTTCCGATCTCCCTATAAGTAGCAGAAATCCG
**HIST1H2BC**	HISTH2B-GSP2-9	GTGACTGGAGTTCAGACGTGTGCTCTTCCGATCTAATGTTTACTTGGAGCTGGTG
**HIST1H2BC**	HISTH2B-GSP2-10	GTGACTGGAGTTCAGACGTGTGCTCTTCCGATCTCAGAAGAAAGATGGCAAGAAG
**HIST1H2BC**	HISTH2B-GSP2-11	GTGACTGGAGTTCAGACGTGTGCTCTTCCGATCTGATGCCAGTGTCGGGATGGAC
**HIST1H2BC**	HISTH2B-GSP2-12	GTGACTGGAGTTCAGACGTGTGCTCTTCCGATCTGCATCTCTTCCAAGGCCATG
**HIST1H2BD**	HISTH2B-GSP2-13	GTGACTGGAGTTCAGACGTGTGCTCTTCCGATCTTGCAACAGTGTTCTAACTATTAACGC
**HIST1H2BD**	HISTH2B-GSP2-14	GTGACTGGAGTTCAGACGTGTGCTCTTCCGATCTGGGATTAGGTGTAAAGATGCTTAC
**HIST1H2BD**	HISTH2B-GSP2-15	GTGACTGGAGTTCAGACGTGTGCTCTTCCGATCTAAGGACGGGAAGAAGCGCAAG
**HIST1H2BD**	HISTH2B-GSP2-16	GTGACTGGAGTTCAGACGTGTGCTCTTCCGATCTAGCTCCCCCGGAAGCAGCAG
**HIST1H2BD**	HISTH2B-GSP2-17	GTGACTGGAGTTCAGACGTGTGCTCTTCCGATCTTCATGAATTCCTTCGTCAACG
**HIST1H2BD**	HISTH2B-GSP2-18	GTGACTGGAGTTCAGACGTGTGCTCTTCCGATCTGTGTCGGGATGGACCTGCTTC
**HIST1H2BE**	HISTH2B-GSP2-19	GTGACTGGAGTTCAGACGTGTGCTCTTCCGATCTGAGGATCAGGAGATGTAGATTTC
**HIST1H2BE**	HISTH2B-GSP2-20	GTGACTGGAGTTCAGACGTGTGCTCTTCCGATCTTGGGTTTACTAAGGCAGTTGC
**HIST1H2BE**	HISTH2B-GSP2-21	GTGACTGGAGTTCAGACGTGTGCTCTTCCGATCTACCAAGGCGCAGAAGAAGGAC
**HIST1H2BE**	HISTH2B-GSP2-22	GTGACTGGAGTTCAGACGTGTGCTCTTCCGATCTAGCTCCCCGGGAAGCAGCAG
**HIST1H2BE**	HISTH2B-GSP2-23	GTGACTGGAGTTCAGACGTGTGCTCTTCCGATCTTGAATTCCTTTGTCAACGACATC
**HIST1H2BE**	HISTH2B-GSP2-24	GTGACTGGAGTTCAGACGTGTGCTCTTCCGATCTGGGGTGGACCTGTTTCAGCAC
**HIST1H2BF**	HISTH2B-GSP2-25	GTGACTGGAGTTCAGACGTGTGCTCTTCCGATCTTTCTCTTTAGGTTGTGGACGAAG
**HIST1H2BF**	HISTH2B-GSP2-26	GTGACTGGAGTTCAGACGTGTGCTCTTCCGATCTCCTTTGGGATTGGGTATGAAG
**HIST1H2BF**	HISTH2B-GSP2-27	GTGACTGGAGTTCAGACGTGTGCTCTTCCGATCTGAAGAAGGATGGTAAGAAGCGCAAG
**HIST1H2BF**	HISTH2B-GSP2-28	GTGACTGGAGTTCAGACGTGTGCTCTTCCGATCTCCTCTGACACGGCGTGCTTAGC
**HIST1H2BF**	HISTH2B-GSP2-29	GTGACTGGAGTTCAGACGTGTGCTCTTCCGATCTCGACACCGGCATCTCATCCAAG
**HIST1H2BF**	HISTH2B-GSP2-30	GTGACTGGAGTTCAGACGTGTGCTCTTCCGATCTGGGGTGGACCTGCTTTAGCAC
**HIST1H2BG**	HISTH2B-GSP2-31	GTGACTGGAGTTCAGACGTGTGCTCTTCCGATCTTTATTCCCTAGAAAACGGTGAAATTC
**HIST1H2BG**	HISTH2B-GSP2-32	GTGACTGGAGTTCAGACGTGTGCTCTTCCGATCTTATTTAAGTGCGTTCATTCTCAC
**HIST1H2BG**	HISTH2B-GSP2-33	GTGACTGGAGTTCAGACGTGTGCTCTTCCGATCTGGATCTCCCTGGAGGTAATGGTCGAG
**HIST1H2BG**	HISTH2B-GSP2-34	GTGACTGGAGTTCAGACGTGTGCTCTTCCGATCTCGCAAGCGCAGTCGTAAGGAGAG
**HIST1H2BG**	HISTH2B-GSP2-35	GTGACTGGAGTTCAGACGTGTGCTCTTCCGATCTTCGGGGTGAACCTGTTTTAG
**HIST1H2BG**	HISTH2B-GSP2-36	GTGACTGGAGTTCAGACGTGTGCTCTTCCGATCTGGGCATCATGAATTCCTTCGTTAACG
**HIST1H2BH**	HISTH2B-GSP2-37	GTGACTGGAGTTCAGACGTGTGCTCTTCCGATCTAGCTGTGCTGTTGAGCCTTC

**HIST1H2BH**	HISTH2B-GSP2-38	GTGACTGGAGTTCAGACGTGTGCTCTTCCGATCTTCCCATAGAATACAGCAGCAC
**HIST1H2BH**	HISTH2B-GSP2-39	GTGACTGGAGTTCAGACGTGTGCTCTTCCGATCTCGCAGAAGAAGGATGGCAAG
**HIST1H2BH**	HISTH2B-GSP2-40	GTGACTGGAGTTCAGACGTGTGCTCTTCCGATCTGGCCTTAGTGCCCTCGGACAC
**HIST1H2BH**	HISTH2B-GSP2-41	GTGACTGGAGTTCAGACGTGTGCTCTTCCGATCTTGAATTCCTTTGTCAACGATAT CTTC
**HIST1H2BH**	HISTH2B-GSP2-42	GTGACTGGAGTTCAGACGTGTGCTCTTCCGATCTTGGAGGAGATGCCGGTGTCG
**HIST1H2BI**	HISTH2B-GSP2-43	GTGACTGGAGTTCAGACGTGTGCTCTTCCGATCTGTGGTCATTTGACGGTATCACT TCGG
**HIST1H2BI**	HISTH2B-GSP2-44	GTGACTGGAGTTCAGACGTGTGCTCTTCCGATCTCCTTGCCAATGCCAAGTCTG
**HIST1H2BI**	HISTH2B-GSP2-45	GTGACTGGAGTTCAGACGTGTGCTCTTCCGATCTCAAGAAGGCGGTGACCAAGGC
**HIST1H2BI**	HISTH2B-GSP2-46	GTGACTGGAGTTCAGACGTGTGCTCTTCCGATCTTCCCTGGAAGTGATGGTCGAG
**HIST1H2BI**	HISTH2B-GSP2-47	GTGACTGGAGTTCAGACGTGTGCTCTTCCGATCTCATTGCAGGCGAGGCTTCCC
**HIST1H2BI**	HISTH2B-GSP2-48	GTGACTGGAGTTCAGACGTGTGCTCTTCCGATCTATCCCCATAGCCTTGGACGAGA TGCC
**HIST1H2BJ**	HISTH2B-GSP2-49	GTGACTGGAGTTCAGACGTGTGCTCTTCCGATCTGTTGAGAGTTTGCAACCAACTC
**HIST1H2BJ**	HISTH2B-GSP2-50	GTGACTGGAGTTCAGACGTGTGCTCTTCCGATCTCTATATAAAAGCGCCTTGTCAT ACCC
**HIST1H2BJ**	HISTH2B-GSP2-51	GTGACTGGAGTTCAGACGTGTGCTCTTCCGATCTACGGCCTTAGTACCCTCGGACA C
**HIST1H2BJ**	HISTH2B-GSP2-52	GTGACTGGAGTTCAGACGTGTGCTCTTCCGATCTCAGAAGAAAGACGGCAAGAAG C
**HIST1H2BJ**	HISTH2B-GSP2-53	GTGACTGGAGTTCAGACGTGTGCTCTTCCGATCTAATGCCGGTGTCAGGGTGGAC
**HIST1H2BJ**	HISTH2B-GSP2-54	GTGACTGGAGTTCAGACGTGTGCTCTTCCGATCTTGGGCATCATGAATTCGTTTGT GAAC
**HIST1H2BK**	HISTH2B-GSP2-55	GTGACTGGAGTTCAGACGTGTGCTCTTCCGATCTTGGGCTTTAAGACGCTTACTTG GC
**HIST1H2BK**	HISTH2B-GSP2-56	GTGACTGGAGTTCAGACGTGTGCTCTTCCGATCTCCGTTTTCTCGATCTGCTGCTC GTC
**HIST1H2BK**	HISTH2B-GSP2-57	GTGACTGGAGTTCAGACGTGTGCTCTTCCGATCTACTTAGCGCTGGTGTACTTGG
**HIST1H2BK**	HISTH2B-GSP2-58	GTGACTGGAGTTCAGACGTGTGCTCTTCCGATCTACTAAGGCGCAGAAGAAGGAC
**HIST1H2BK**	HISTH2B-GSP2-59	GTGACTGGAGTTCAGACGTGTGCTCTTCCGATCTTAGAGGAGATGCCGGTGTCG
**HIST1H2BK**	HISTH2B-GSP2-60	GTGACTGGAGTTCAGACGTGTGCTCTTCCGATCTCCTCTAAGGCCATGGGAATC
**HIST1H2BL**	HISTH2B-GSP2-61	GTGACTGGAGTTCAGACGTGTGCTCTTCCGATCTGGGTTGGACAAGAGCTTGAG
**HIST1H2BL**	HISTH2B-GSP2-62	GTGACTGGAGTTCAGACGTGTGCTCTTCCGATCTCCCACTACCTCGGCCGCATAAA C
**HIST1H2BL**	HISTH2B-GSP2-63	GTGACTGGAGTTCAGACGTGTGCTCTTCCGATCTGCACGGCGGTCTGGATCTCC
**HIST1H2BL**	HISTH2B-GSP2-64	GTGACTGGAGTTCAGACGTGTGCTCTTCCGATCTGTGACCAAGGCCCAGAAGAAG GATG
**HIST1H2BL**	HISTH2B-GSP2-65	GTGACTGGAGTTCAGACGTGTGCTCTTCCGATCTTGGCCTTAGAAGAGATGCCGG TGTC
**HIST1H2BL**	HISTH2B-GSP2-66	GTGACTGGAGTTCAGACGTGTGCTCTTCCGATCTTCTTCTAAGGCCATGGGAATC
**HIST1H2BM**	HISTH2B-GSP2-67	GTGACTGGAGTTCAGACGTGTGCTCTTCCGATCTAGAGGACGAAACAGCCCTAAG
**HIST1H2BM**	HISTH2B-GSP2-68	GTGACTGGAGTTCAGACGTGTGCTCTTCCGATCTGTCACGGCGGAACTGTTACTGC
**HIST1H2BM**	HISTH2B-GSP2-69	GTGACTGGAGTTCAGACGTGTGCTCTTCCGATCTTCTGGATCTCCCTCGAAGTGAT GGTC
**HIST1H2BM**	HISTH2B-GSP2-70	GTGACTGGAGTTCAGACGTGTGCTCTTCCGATCTCAACGACATCTTTGAGCGTATC G
**HIST1H2BM**	HISTH2B-GSP2-71	GTGACTGGAGTTCAGACGTGTGCTCTTCCGATCTGCCTTGGAAGAGATGCCGGTG TC
**HIST1H2BN**	HISTH2B-GSP2-72	GTGACTGGAGTTCAGACGTGTGCTCTTCCGATCTCCTCCAATTTTCCGGCAGTTAC
**HIST1H2BN**	HISTH2B-GSP2-73	GTGACTGGAGTTCAGACGTGTGCTCTTCCGATCTCCGAGACTGACCGAACGTTCC G
**HIST1H2BN**	HISTH2B-GSP2-74	GTGACTGGAGTTCAGACGTGTGCTCTTCCGATCTGCCCAGAAGAAGGACGGCAAG
**HIST1H2BN**	HISTH2B-GSP2-75	GTGACTGGAGTTCAGACGTGTGCTCTTCCGATCTGGCTCACTTGGAACTGGTGTAC TTGG
**HIST1H2BN**	HISTH2B-GSP2-76	GTGACTGGAGTTCAGACGTGTGCTCTTCCGATCTTTCGTCAATGACATCTTCGAG

**HIST1H2BN**	HISTH2B-GSP2-77	GTGACTGGAGTTCAGACGTGTGCTCTTCCGATCTGGCCTTGGACGAGATACCGGTGTC
**HIST1H2BO**	HISTH2B-GSP2-78	GTGACTGGAGTTCAGACGTGTGCTCTTCCGATCTATCTGAGTAACTTCCAATCAGAC
**HIST1H2BO**	HISTH2B-GSP2-79	GTGACTGGAGTTCAGACGTGTGCTCTTCCGATCTACCCAAAAGACTACAAGCAAAATG
**HIST1H2BO**	HISTH2B-GSP2-80	GTGACTGGAGTTCAGACGTGTGCTCTTCCGATCTGCAGCCGCAAAGAGAGTTACTC
**HIST1H2BO**	HISTH2B-GSP2-81	GTGACTGGAGTTCAGACGTGTGCTCTTCCGATCTCGTCAATGACATCTTTGAGCGCATCG
**HIST1H2BO**	HISTH2B-GSP2-82	GTGACTGGAGTTCAGACGTGTGCTCTTCCGATCTGTCGGGGTGGACTTGCTTCAGC
**HIST2H2BE**	HISTH2B-GSP2-83	GTGACTGGAGTTCAGACGTGTGCTCTTCCGATCTAAGAGCCTTTGGAGTCAAGC
**HIST2H2BE**	HISTH2B-GSP2-84	GTGACTGGAGTTCAGACGTGTGCTCTTCCGATCTAATCAGGCCCGCCCATTCTCTTAC
**HIST2H2BE**	HISTH2B-GSP2-85	GTGACTGGAGTTCAGACGTGTGCTCTTCCGATCTGTCTGGATCTCGCGGGATGTGATGG
**HIST2H2BE**	HISTH2B-GSP2-86	GTGACTGGAGTTCAGACGTGTGCTCTTCCGATCTAAGAGAGCTACTCCATCTACG
**HIST2H2BE**	HISTH2B-GSP2-87	GTGACTGGAGTTCAGACGTGTGCTCTTCCGATCTGAGAGCTACTCCATCTACGTGTAC
**HIST2H2BF**	HISTH2B-GSP2-88	GTGACTGGAGTTCAGACGTGTGCTCTTCCGATCTGGTGGTTGATCTATTGCGTCCCTTGC
**HIST2H2BF**	HISTH2B-GSP2-89	GTGACTGGAGTTCAGACGTGTGCTCTTCCGATCTGGCTCTATAAGTAGCGCATAACCAG
**HIST2H2BF**	HISTH2B-GSP2-90	GTGACTGGAGTTCAGACGTGTGCTCTTCCGATCTGTCTGGATCTCGCGGGATGTGATGG
**HIST2H2BF**	HISTH2B-GSP2-91	GTGACTGGAGTTCAGACGTGTGCTCTTCCGATCTGAAAGTGCAGAAGAAGGACGG
**HIST2H2BF**	HISTH2B-GSP2-92	GTGACTGGAGTTCAGACGTGTGCTCTTCCGATCTTCCGTTTACGTGTACAAGGTG
**HIST3H2BB**	HISTH2B-GSP2-93	GTGACTGGAGTTCAGACGTGTGCTCTTCCGATCTAGCCTTCATTTGAATACAAAACGTAC
**HIST3H2BB**	HISTH2B-GSP2-94	GTGACTGGAGTTCAGACGTGTGCTCTTCCGATCTGGTTCAGGACGCCGAGGAACG
**HIST3H2BB**	HISTH2B-GSP2-95	GTGACTGGAGTTCAGACGTGTGCTCTTCCGATCTGCACAGAAGAAGGACGGCAAG
**HIST3H2BB**	HISTH2B-GSP2-96	GTGACTGGAGTTCAGACGTGTGCTCTTCCGATCTACTTGGTGACAGCCTTGGTG
**HIST3H2BB**	HISTH2B-GSP2-97	GTGACTGGAGTTCAGACGTGTGCTCTTCCGATCTTTCGTCAATGACATCTTCGAG
**HIST3H2BB**	HISTH2B-GSP2-98	GTGACTGGAGTTCAGACGTGTGCTCTTCCGATCTATGTCATTGACGAAGGAGTTC

Supplementary Table 6

Primers for H2B genotyping

Gene	Forward	Reverse
**HIST1H2BB**	CATAAACCCCACCCCTCAGT	TGTCTTCGCTAACATTCCAGT
**HIST1H2BC**	CACGGATGACAACTGTGCAG	GGCCACAGCTCTTTTAGTGG
**HIST1H2BD**	ACATTGGCATTTGTGACGACA	GGTACTGTGGTGCTAAGTCCA
**HIST1H2BE**	GCATGCAGACTTCACGACAA	GCCTATTTCAGCTGCAGGAG
**HIST1H2BF**	TGCCAGTTTACGATAGGAGC	AGCCTTTGGGATTGGGTATG
**HIST1H2BG**	TGCGTCACTAACACATTGCC	AAACTGGTCTCGATCCGCA
**HIST1H2BH**	GACCTACAAAAACGGCCAGC	GTATTGGCGACACTTCTCCC
**HIST1H2BI**	TAATGAGGGCGTTTGGGCTC	ACAACATGGGTGGCTCTTAG
**HIST1H2BJ**	CTACCAATCAGACACAAGAC	AGGAATACAAGCACCAGCTC
**HIST1H2BK**	GCCAGACTCGATTACAAGCA	TGACCTCTGACGTTACCCTG
**HSIT1H2BL**	GGCATCTGTCGTCATCCACG	GAAGAGCGAACCAGCTCTTC
**HT1H2BM**	GCAAAACTGGCATCTGACGT	ATGTACGAAGCGAGTCCCAA
**HSIT1H2BN**	TGGTTAAACGCACAACTTCATTC	ACCGGGATTAGAGGCTTGAG
**HIST1H2BO**	ACGTCATCTGAGTAACTTCCAA	TTACCTTTTCCCGCCGTCTT
**HIST2H2BE**	ATGAACGACTTTCGGAGCCC	TGATTAGGTGGGTGGCTCTG
**HIST2H2BF**	AGTTAACCAATGAAAGCGCAG	CCTAAACCCGAATGCATCCG
**HIST3H2BB**	GCGTTCGATTGGATGGCTAT	ACCTGAGCAGTTTCTACCCC

Primers for CRISPR/Cas9 knockin cells genotyping

Primer name	Forward	Reverse
**Pair 1**	GTCTCGTTATCAGGGTGGTC	CATCGTCTTTGTAATCCTTG
**Pair 2**	AGTAACTTCCAATCAGACAG	AGCGTCTATACTCACACGCAAA
**Pair 3**	GCCTTCTTGACGAGTTCTTC	CCTCCTATTTCTGTGATCTC

Primers for off-target analysis of the CRISPR/Cas9

Target locus	Forward	Reverse
**chrY:13310607**	TGTGTGTGGAGATTGGAGTG	CAGACTGGTCTCTGCATTCC
**chr12: 123983633**	ATGGCAGATGGAAGGGTGTC	CCTGGAAACTTCAAGTCTTCAC
**chr15: 59447921**	CCTGCTCAAGGTCGGAAACTT	AAGGGCATTTCTTTAGTCTCTGGA
**chr1: 161369988**	AGATAGTTGGGCAGGGCTCC	ACCTAGCTTCAGTCTCTTCATC
**chr17: 18554732**	CCATGCAACTTCAACATATAG	TGACACATTTTCTTGCTCTC
**chr5: 43529893**	GTGTTGGGATTACAGGCACG	GGGTGATTGGTCATTTCATGTG
**chr1: 5495269**	TACTCATGCATGCATTTGCC	GCTTCTGCAAGCACAGAATG
**chr11: 125074688**	ACATTCCCGGTCAGAGACAG	TCTGGGAGTGTACGGTCTTG
**chr18: 66309528**	TCGTCGTGCACTTGGCATTG	CAAGCATCATCAGGTCAACAG
**chr10: 112255820**	ACAGAGCCGCCTCAGCGATG	GAATGAATTGTAAGCCAGGCTGC
**chr1:227173781**	CACTCGCACTGACTCGATTC	AACACGAGGGTAACATGGAC
**chr17: 27222984**	GATGACCATGAGATGTATTAGC	GGAGATCTTGCTGAGAACTG
**chr11: 63763805**	GAAGTTCTCTTGGGCTGAAAG	ACAGGTTAACATCAGGCCAG
**chr4:129149438**	AGAGAATTGCTGAGCAGGGC	CCAGTGTGAGCAAAGGAGAC
**chr1: 5041357**	AATGTGCAGCCAGGAAGTGG	GTATAGCTATAGTCATTGGTGC
**chr9:34363031**	CAGACTATGTATATCCCTGTGC	AGGAGATGCTCTGGAGCTTG
**chr:734363031&7102256981**	GTGAGCCGCGGATCGCTGAC	GCGATGGCCAAGCGCAGCTC
**chr10: 82259029**	ATCATGGGTGAGAGGCTGCC	CAGTGAGACCACGCTGTGTC
**chr5: 1239117**	AGCCTGCACGGCTTCTAGAG	AGGCGCACATGAGAACGTAG

Supplementary Methods

Histone H2B targeted sequencing

Pancreatic cancer specimens were obtained from subjects enrolled at the Baylor University Medical Center. A written informed consent was obtained from all patients prior to participating in the study and the study was approved by the Institutional Review Board at Baylor Scott & White Health (IRB, 015-196). DAN from these specimens was extracted using Qiagen's DNA Easy kit, using instructions provided by the manufacturer. Target sequencing of 17 histone genes were performed by using the AMP method with modifications [1]. Briefly, the genomic DNA was subjected to end-repair and A-tailing (KAPA HyperPrep Kit, Roche) at 37°C for 15 min, followed by 65°C for 15 min and ligation (KAPA HyperPrep Kit, Roche) with a universal half-functional adapter (**Supplementary Table 5**) at 16°C for 30 min, followed by 30°C for 30 min. SPRI-cleaned ligated libraries were subjected to two rounds of nested PCR at 20 cycles each for target enrichment (Platinum Taq Polymerase, Life Technologies). The first round of PCR was performed using a first pool of ninety-eight HistH2B target specific primers (IDTDNA, (**Supplementary Table 6**, GSP1) with the following thermal cycling condition: 95°C for 5 min; 20 cycles of (95°C for 30 sec; 72°C for 1 sec; ramping down at 0.2^{o}C/sec; 60°C for 5 min; ramping up at 0.2^{o}C/sec; 72°C for 1 sec); 4°C hold. After SPRI cleanup, a second round of PCR was conducted using a second pool of 3' nested HistH2B target specific primers (IDTDNA, (**Supplementary Table 7**, GSP2) downstream of respective initial first pool target primers, and P558 and i7 index primer, with the following thermal cycling condition: 95°C for 5 min; 20 cycles of (95°C for 30 sec; 60°C for 5 min); 4°C hold. The final sequencing libraries were quantified by KAPA Library Quantification Kits for Illumina and sequenced on Illumina HiSeq System according to manufacturer's protocol. Data de-multiplexing of Index 1 was performed by Illumina bcl2fq v2.19, followed by custom scripts for Index 2 demultiplexing, adaptor trimming, and unique molecular identifier (UMI) parsing. FASTQ sequences were aligned to human reference genome (hg19) using BWA MEM [2], and variant calling were performed using a UMI-aware custom script. Of 125 samples sequenced, four samples failed QC (Percentage of all targeted bases having at least 500X UMI-unique read depth [%>500X]: 44.6%, 61.1%, 69.3% and 69.7%). The remaining 121 samples were sequenced to a mean UMI-unique read depth of 6,627X, and a mean %>500X at 91.2% (range 70.3% - 100%).

Histone gene mutation screening analysis in TCGA

All 32 cancers' mutation data in TCGA database was downloaded from Broad GDAC Firehose by 'RTCGAToolbox' package (accessed on Nov 1, 2015) [3]. Basic statistical analysis was performed to summarize all missense mutations on histone genes among all cancer types.

Histone purification and nucleosome loading

The human WT histones (H2A, H2B, H3, and H4), linker histone H1.0, and H2BG53D were cloned into pET11a (Merck). These constructs were expressed in BL21-CodonPlus (DE3)-RIL competent cells (Agilent), and they were purified from the inclusion bodies. The histone mixture was resuspended in unfolding buffer (20 mM Tris, pH 7.5; 7 M guanidine hydrochloride; 4 mM DTT) and the mixture was dialyzed against water at 4°C for 4 hours, then dialyzed against refolding buffer (50 mM Tris, pH 7.5; 2 M NaCl; 0.5 mM EDTA; 5 mM 2-mercaptoethanol) overnight to get histone octamer. The histone octamer was further mixed with 258 bp DNA containing the 601 nucleosome positioning sequence in a solution containing 2 M NaCl. The nucleosomes were then reconstituted by the salt-dialysis method. For the optical tweezers'

nucleosome assay, recombinant H1.0 was added during the nucleosomes loading and loaded nucleosomes were further purified by polyacrylamide gel electrophoresis (Prep Cell, Bio-rad).

Single-molecule optical tweezers' nucleosome stability assay

For quantifying nucleosome stability at the single-molecule level, purified 258 bp nucleosome was ligated with 0.8 kb and 0.5 kb lambda DNA that were obtained by PCR using primers containing biotin or digoxygenin (BGI), respectively. These molecules allow the formation of a DNA tether containing a single nucleosome between the streptavidin and anti-digoxygenin beads held by the optical trap and micropipette, respectively. To measure the nucleosome inner unfolding and refolding force, the tension was increased by stepping the trap at 100 nm/s in the buffer (20 mM Tris-HCl, pH 7.5; 300 mM NaCl). The data were collected at 200 Hz and decimated to 40 Hz to extract the force. To extract the nucleosome wrapping and unwrapping rates at the outer wrap, the beads were held at constant positions in the low salt buffer (10 mM Tris, pH 7.5; 10 mM NaCl). The data were collected at 1 kHz and decimated to ~250 Hz. Transitions were determined by running a t-test analysis between two adjacent windows of the wrapping/unwrapping traces. The energy barrier of the outer wrap was extracted by the methods shown in [4-6].

Purification of mammalian RNA polymerase II

Pol II was purified as described [7] with modifications. Generally, 100 g of fresh pig liver was broken into small pieces and homogenized in 200 ml of chilled Buffer A (50 mM Tris-HCl, pH 7.9; 0.1 mM EDTA; 10 μM ZnCl2; 1 mM MgCl2; 5 mM BME; 1 mM PMSF; 10% glycerol (v/v)). The material was then subjected to sonication on ice and centrifuged at 11,000 g for 30 min at 4°C. Polyethyleneimine (PEI) was added to the lysate to a final concentration of 0.15% (w/v). After stirring for 10 min at 4°C, the material was centrifuged at 4°C 300 g for 20 min. The PEI pellet was re-suspended and extracted with 500 ml of Buffer B (50 mM Tris-HCl, pH 7.9; 0.1 mM EDTA; 10 μM ZnCl2; 1 mM MgCl2; 5 mM BME; 1 mM PMSF; 250 mM (NH4)2SO4; 10% glycerol (v/v)) for 30 min at 4°C with stirring. The material was then clarified by centrifugation at 11,000 g for 40 min. Lysate was diluted by Buffer A to a final concentration of 150 mM (NH4)2SO4 and loaded onto a 100 ml Q-Sepharose column, which was washed by three column volumes of Buffer C (50 mM Tris, pH 7.9; 0.1 mM EDTA; 10 μM ZnCl2; 1 mM MgCl2; 5 mM BME; 1 mM PMSF; 150 mM (NH4)2SO4; 10% glycerol (v/v)) and then eluted with Buffer D containing 500 mM (NH4)2SO4. The eluent was then purified by a Heparin-affinity chromatography column. Elution was achieved with a linear gradient of 210-650 mM (NH4)2SO4 in Buffer A. The eluent was finally purified by 8WG16 antibody (Abcam) immunoprecipitation, which recognizes the C-terminal domain of the largest subunit of Pol II.

Pol II Transcription elongation assay with nucleosomal DNA template

0.5 μg purified Pol II (1 pmol) was immobilized on 8WG16-Agarose Protein A beads. RNA and template DNA (TDS) scaffold was assembled by annealing the template strand DNA (3 pmol) and the [γ-32P]-ATP end-labeled 14 mer RNA (3 pmol) by linear gradient from 42°C to room temperature [8,9]. Transcription elongation complex (TEC) was formed by incubating the scaffold with RNAPII coupled agarose at room temperature for 10 min followed by the addition of non-template strand (NDS, 9 pmol) at 37°C for 10 min. The TEC was washed with TB40 (20 mM Tris, pH 7.9; 5 mM MgCl2; 5 mM BME; 10 μM ZnCl2; 40 mM KCl) and ligated to the 601R (601 Reverse DNA template) nucleosome through DraIII site at 16°C for 1 hour. Then the complex was

transcribed in the presence of the indicated concentrations of KCl and 500 μM rNTPs. All transcription reactions were stopped by adding 1 mg proteinase K and incubated at 30°C for 20 min. Urea stop buffer (89 mM Tris, pH 8.0; 8 M urea; 89 mM boric acid; 50 mM EDTA; 0.25% bromophenol blue) was further added and the reactions were incubated at 65°C for 20 min. Transcripts were resolved on 12% denaturing polyacrylamide gels. Gels were scanned and quantified by a Typhoon phosphorimager (GE healthcare).

DNA damage assays
Cells were treated with either MMS (1:10000, Sigma) or Camptothecin (2 μM, Goldbio) for 1 hour. The cells were then washed twice before recovery for indicated time points before whole cell lysates were collected for immunoblot analysis or fixed for immunofluorescence staining.

Immunofluorescence staining
Cells seeded on TEK chambers were fixed with 3% PFA for 12 min. The cells were washed twice with PBS before permeabilization with 0.5% Triton Solution (20 mM HEPES pH 7.4; 50 mM NaCl; 3 mM MgCl2; 300 mM Sucrose; 0.5% Triton X-100) for 5 min. The cells were then incubated in blocking buffer (5% normal goat serum/0.1% Triton X-100 in PBS), followed by sequential staining with primary and fluorescent secondary antibodies. Each staining was performed for 20 min at 37°C. Lastly, the nuclei were stained with DAPI (Invitrogen) before mounting the samples in Prolong Gold Antifade Mountant (Thermo). The cells were imaged using a 20X objective on a Nikon Eclipse Ni-E upright fluorescence microscope. The images were analyzed using ImageJ. Average number of γH2Ax and 53BP1 foci per cell were calculated from 100 cells per sample.

CRISPR-Cas9 knockin the H2BG53D mutation in pancreatic cells
S2VP10 was maintained in DMEM supplemented with 10% FBS. A repair template and lentiCRISPRv2 co-expressing the sgRNA and HA-tagged Cas9 were transfected to S2VP10 on 10 cm plate using PEI (DNA: PEI = 1:6) (polyscience, 23966). Transfected cells were then split 1:4 24 hours post-transfection. 2.5 mg/ml G418-sulphate (Gold Biotechnology, G-418) was added to the medium one day after the split. Cells were kept in culture until single colonies formed. Clones were then pick to 24-well plate for expansion and collection of genomic DNA for PCR genotyping.

Cell fractionation assay
Cells were collected by trypsin and and washed twice with cold 1XPBS before lysing in Buffer A (10 mM HEPES, pH7.9; 10 mM KCl; 1.5 mM MgCl2; 0.34 M sucrose; 10% glycerol; 1 mM DTT; 0.1% Triton X-100; protease inhibitor cocktail) on ice for 8 min. S1 and P1 were obtained by centrifuging the lysate at 1300g, 4°C for 5 min. S1 was clarified by centrifuging at 20,000g, 4°C for 5 min, supernatant was collected as S2. P1 was washed thrice with Buffer A (without TritonX-100) before lysing in Buffer B (3 mM EDTA; 0.2 mM EGTA; 1 mM DTT; protease inhibitor cocktail) for 30 min. S3 and P3 was obtained by centrifuging the lysate at 1700g, 4°C for 5 min. P3 was then washed twice with Buffer B. The pellet was resuspended in PBS and sonicated with Diagenode bioruptor (30 sec on/off, 6 cycles, high power).

BrdU labeling
Cells were incubated with 10 μM of BrdU for 1 hour. The cells were washed with PBS three times 2 min before fixing with 3% formaldehyde for 12 min at room temperature. The fixed cells were

permeabilized with 0.5% Triton solution (20 mM HEPES pH 7.4; 50 mM NaCl; 3 mM MgCl2; 300 mM Sucrose; 0.5% Triton X-100) for 5 min before treatment with 2N HCl for 20 min at room temperature. After washing with PBS, the cells were subjected to immunofluorescence staining with an anti-BrdU antibody.

Cell proliferation assay

3000 cells were seeded to each well of a 96-well plate. Medium containing 10% CCK-8 solution (Dojindo, CK04) was incubated with the cells for 2.5 hours prior to OD450 measurement (BioTek Synergy™ H1 Microplate Reader). Proliferation rate was assayed at 24, 48, and 72 hours after cell seeding.

Transwell migration assay

25,000 cells resuspended in 1% FBS DMEM were seeded to the top chamber (Corning® Costar® Transwell® cell culture inserts, PET membrane, pore size 8.0 µm). DMEM containing 10% FBS was added to the lower chamber as a chemoattractant. Cells were allowed to migrate for 48 hours before crystal violet staining.

Gap closure assay

A cell free gap was created using a 2-well silicone insert (Ibidi, 80241). 25,000 cells were seeded to each well of the insert. The insert was removed 18 hours after cell seeding. Images of the gap were taken immediately after the removal of the insert and 18 hours after the removal.

References

1 Zheng, Z. et al. Anchored multiplex PCR for targeted next-generation sequencing. Nat Med 20, 1479-1484, doi:10.1038/nm.3729 (2014).

2 Li, H. & Durbin, R. Fast and accurate short read alignment with Burrows–Wheeler transform. bioinformatics 25, 1754-1760 (2009).

3 Samur, M. K. RTCGAToolbox: a new tool for exporting TCGA Firehose data. PLoS One 9, e106397, doi:10.1371/journal.pone.0106397 (2014).

4 Mahat, D. B. et al. Base-pair-resolution genome-wide mapping of active RNA polymerases using precision nuclear run-on (PRO-seq). Nature Protocols 11, 1455-1476, doi:10.1038/nprot.2016.086 (2016).

5 Bustamante, C., Chemla, Y. R., Forde, N. R. & Izhaky, D. Mechanical processes in biochemistry. Annu Rev Biochem 73, 705-748, doi:10.1146/annurev.biochem.72.121801.161542 (2004).

6 Liphardt, J., Onoa, B., Smith, S. B., Tinoco, I., Jr. & Bustamante, C. Reversible unfolding of single RNA molecules by mechanical force. Science 292, 733-737, doi:10.1126/science.1058498 (2001).

7 Hu, X. et al. A Mediator-responsive form of metazoan RNA polymerase II. Proc Natl Acad Sci U S A 103, 9506-9511, doi:10.1073/pnas.0603702103 (2006).

8 Palangat, M. et al. Efficient reconstitution of transcription elongation complexes for single-molecule studies of eukaryotic RNA polymerase II. Transcription 3, 146-153, doi:10.4161/trns.20269 (2012).

9 Kireeva, M. L., Lubkowska, L., Komissarova, N. & Kashlev, M. Assays and affinity purification of biotinylated and nonbiotinylated forms of double-tagged core RNA polymerase II from Saccharomyces cerevisiae. Methods Enzymol 370, 138-155, doi:10.1016/S0076-6879(03)70012-3 (2003).
